# Simple strategies for semi-supervised feature selection

**DOI:** 10.1007/s10994-017-5648-2

**Published:** 2017-07-17

**Authors:** Konstantinos Sechidis, Gavin Brown

**Affiliations:** School of Computer Science, University of Manchester, Manchester, M13 9PL UK

**Keywords:** Semi-supervised, Positive unlabelled, Feature selection

## Abstract

What is the simplest thing you can do to solve a problem? In the context of semi-supervised feature selection, we tackle exactly this—how much we can gain from two simple *classifier-independent* strategies. If we have some binary labelled data and some unlabelled, we could assume the unlabelled data are all positives, or assume them all negatives. These minimalist, seemingly naive, approaches have not previously been studied in depth. However, with theoretical and empirical studies, we show they provide powerful results for feature selection, via hypothesis testing and feature ranking. Combining them with some “soft” prior knowledge of the domain, we derive two novel algorithms (*Semi*-JMI, *Semi*-IAMB) that outperform significantly more complex competing methods, showing particularly good performance when the labels are missing-not-at-random. We conclude that simple approaches to this problem can work surprisingly well, and in many situations we can provably recover the exact feature selection dynamics, *as if we had labelled the entire dataset*.

## Introduction

Many real-world applications have limited access to *labelled* data, but abundant access to large amounts of *unlabelled* data. Our work focuses on two *semi-supervised* scenarios that occur in binary problems: when the labelled set contains *both* positive and negative examples, and, a more restricted version, when *only* positive examples are labelled (also known as *positive-unlabelled* learning). An important research direction is to transfer techniques and methodologies from supervised learning over to such semi-supervised situations.

An easy solution is simply to *ignore* the unlabelled data; but this point, of when unlabelled data in fact may help, is a controversial, and challenging question (Singh et al. 2009; Li and Zhou 2015). Some studies in the literature focused on providing answers on how unlabelled data are beneficial and *guarantee* improvements in solving *classification problems* (Sokolovska 2008; Balcan and Blum 2010; Krijthe and Loog 2015; Loog 2016). Our work focuses on using unlabelled data to solve *feature selection problems*. We note that there are *two intimately related research questions*, often conflated, and as we will see, it is beneficial to disentangle them. These concern the *testing* and *ranking* of features, in relation to the label.

- Q1. Testing:*“Is there a* *significant* *dependency between feature* *X* *and label* *Y*?”
- Q2. Ranking:*“Using a finite sample of data, can we recover a* *ranking* *of features, that would be close to that we would obtain if we had access to the true distribution?”*

We focus on *filters* for feature selection, allowing *classifier-independent* answers to these questions—in particular with information theoretic methods. This is as opposed to *classifier-dependent* wrapper/embedded methods (Guyon et al. 2006). Our goal is therefore: approaches for semi-supervised information theoretic feature selection—as such, this can be seen as a semi-supervised companion to Brown et al. (2012).

**Fig. 1:**
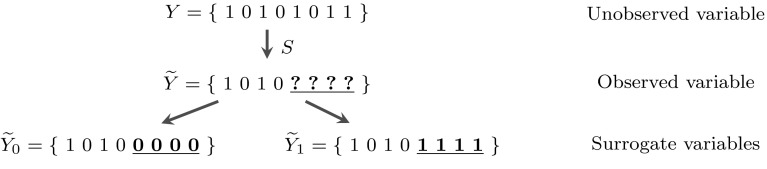
Illustration of the two simple strategies we investigate. From the (unobservable) true labels *Y*, we assume an unknown process *S* which generates $\widetilde{Y}$, with missing values. Now, we have two simple (and clearly incorrect) *inference-free* actions—assume all unlabelled objects are negative (*left branch*) or all positive (*right branch*). The questions tackled in this paper concern what happens if we use ${\widetilde{Y}_0}$ or $\widetilde{Y}_1$ instead of *Y*, for hypothesis testing or feature ranking

In terms of data, we tackle two semi-supervised scenarios—when the labels are missing completely at random (MCAR), and a missing-not-at-random scenario (MAR-C) where the class labels are missing according to a mechanism, *dependent on the class label itself* (Moreno-Torres et al. 2012). The latter might occur for example when there is a social stigma associated with reporting of a label, such as income levels or HIV incidence. Our aim is to deeply understand two very simple (but commonly adopted) strategies that are *inference-free*. They are simply: we assume all missing labels are in fact negative, or assume they are in fact positives (Fig. 1). Either route results in a “surrogate” variable, ${\widetilde{Y}_0}$ or $\widetilde{Y}_1,$ used *in place of* the unobservable *Y*,  after which we proceed with feature selection *exactly as if we had a usual, fully-supervised, scenario*.

Clearly, both of these are highly likely to be false assumptions, but they are popular. For example, Elkan and Noto (2008, Sect. 2) use them to learn classifiers from positive unlabelled data, while Blanchard et al. (2010, Sect. 3) for semi-supervised novelty detection. What is most surprising, is that these have very similar statistical properties to the (unobservable) full label vector. We use these properties to derive novel feature selection algorithms, which turn out to be highly competitive with significantly more complex procedures.

### Summary of results

We now present, in summary, the results of the work.

**Results on Semi-Supervised Hypothesis Testing**

For hypothesis testing, we use the *G*-test of independence—such a test is characterised by its false positive rate (FPR) and false negative rate (FNR). The first contribution is in terms of asking what happens to the FPR/FNR if we test with surrogate variables ${G}(X;{\widetilde{Y}_0})$ or $G(X;\widetilde{Y}_1)$, instead of the ideal (unobservable) *G*(*X*; *Y*)? In Sect. 3 we prove that the answer to this question is:

- Both surrogate tests will have *exactly the same* FPR as the ideal test.
- Both surrogate tests will have a *higher* FNR than the ideal test.

This result turns out to be true regardless of the data missingness scenario: MCAR or MAR-C. The higher FNR is clearly an undesirable consequence so we offer two solutions, that become possible if the user is able to provide some belief over the true underlying class probability $p(y=1)$.

The first solution, in case the user has the luxury of collecting more samples, is a “correction factor” that specifies the minimum number of new samples necessary to achieve a desirable FNR. In case this is not possible, the second solution is a simple “switching threshold” (Definition 3) that tells the user *which one* of the two surrogates will have the lesser FNR. If the user believes the true class probability is below the threshold, they should use ${\widetilde{Y}_0}$, i.e. assume all missing labels are negative, otherwise, they should use $\widetilde{Y}_1$. In pseudocode:
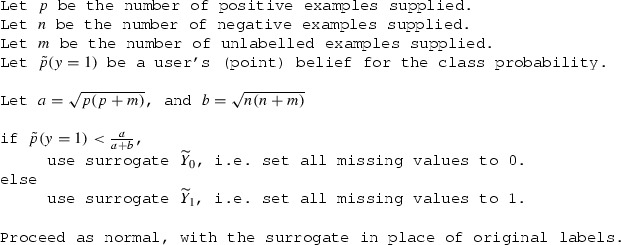
 Our proposal for semi-supervised feature selection is to apply the above procedure. In the following sections we will show significant empirical evidence that this is a surprisingly powerful approach. This can be used (for example) in the hypothesis testing phase of Markov Blanket discovery, using the IAMB algorithm (Tsamardinos and Aliferis 2003), but with *semi-supervised nodes* in the Bayesian network—we refer to this as *Semi*-IAMB.^1^

**Results on Semi-Supervised Feature Ranking**

Here the question we address is: if we were to *rank* features by their estimated mutual information with a surrogate label, i.e. use $\hat{I}(X;{\widetilde{Y}_0})$ or $\hat{I}(X;\widetilde{Y}_1)$, which one will provide a closer feature ranking to that of the true unobservable $\hat{I}(X;Y)$?

A theoretical analysis in Sect. 4 shows the answer to this is:

- In the limit of large data, both surrogates produce *exactly* the same ranking as the unobservable $\hat{I}(X;Y)$.
- With finite samples, the same switching threshold as before can be used to find which will produce a closer ranking. If the user believes the class probability is below the threshold, then they should rank features by $\hat{I}(X;{\widetilde{Y}_0})$, otherwise they should use the other surrogate.

The same results apply to conditional mutual information terms, and hence to the various information theoretic criteria reviewed in Brown et al. (2012). As before, we apply the pseudocode as above to pick which surrogate we will use, then apply one of the selection criteria. For example, one can apply the JMI feature selection criterion (Yang and Moody 1999) after our procedure, which we then refer to as *Semi*-JMI.

Sections 5 and 6 present extensive empirical studies (11 datasets, 7 competing methods), and the proposed approach is shown to be competitive on several evaluation metrics. Furthermore, in controlled experiments, by varying the missingness scenario smoothly from MCAR to MARC (i.e. toward more label-biased data), we show that our method dominates the others.

Parts of this article have been published in two previous conference papers: Sects. 3.4 and 5 in Sechidis and Brown (2015), while parts of Sect. 3.3 in Sechidis et al. (2014). Those two previous works focused only on feature selection through hypothesis testing. Here we demonstrate these results in a framework for information feature selection through *testing* and *ranking*, by giving a more extended theoretical analysis (Sects. 3, 4) and additional results for different semi-supervised scenarios with novel experiments (Sect. 6).

## Background

In this section we will give the background material for our work. Firstly we will review information theoretic feature selection, via hypothesis testing and ranking. Then we will formally introduce the semi-supervised settings that we will focus on, and we will motivate our approach to solve the feature selection problem in these settings. Throughout this work we focus on information theoretic testing and estimation by exploring a known relationship between the maximum likelihood estimator of the mutual information and the *G*-test of independence. Appendix A provides a tutorial on hypothesis testing and estimation of mutual information.

### Feature selection by testing independence—Markov Blanket discovery

In fully-labelled scenarios we observe a sample dataset $\{ {\mathbf{x}}^i, y^i \}_{i=1}^N$. The feature vector ${\mathbf{x}} = [x_1 \ldots x_d]$ is a realization of the joint random variable ${\mathbf{X}} = X_1\ldots X_d,$ and, without loss of generality, we assume categorical features.^2^ With a slight abuse of notation, in the rest of our work, we interchange the symbol for a set of variables and for their joint random variable.

Feature selection is a challenging problem, not only to solve, but also to define the concept of “optimal” feature set—after all, one feature set may work well with one classifier, but not with another. In the special case where the data can be assumed to be a sample from an unknown Bayesian network, the optimal feature set is uniquely defined, and known as the *Markov Blanket* (MB) of a target variable. Pearl (1988) formally defined the MB of a variable *Y* as the set of features ${\mathbf{X}_{\scriptscriptstyle {\mathrm{MB}}}}$ with the property  for every ${\mathbf{Z}}\subseteq \mathbf{X} \backslash {\mathbf{X}_{\scriptscriptstyle {\mathrm{MB}}}}$. In probabilistic graphical models terminology, the target variable *Y* becomes conditionally independent from the rest of the graph $\mathbf{X} \backslash {\mathbf{X}_{\scriptscriptstyle {\mathrm{MB}}}}$ given its MB ${\mathbf{X}_{\scriptscriptstyle {\mathrm{MB}}}}$.

Koller and Sahami (1996) showed that the MB of a target variable is the optimal set of features to minimize the amount of predictive information lost during feature selection, since it minimizes $D_{KL}(p(y|{\mathbf{x}})||p(y|{\mathbf{x}}_{MB}))$. In this context, discovering the MB can be useful for eliminating irrelevant features or features that are redundant in the context of others, and as a result it plays a fundamental role in filter feature selection. There are many different approaches to derive the MB from finite datasets. In our work we will will use the Incremental Association Markov Blanket (IAMB) (Tsamardinos and Aliferis 2003) algorithm, which can be seen in Algorithm 1. IAMB consists of two-stages: *growing*, where we add features to the Candidate Markov Blanket (CMB) set until the remaining features become independent of the target given the candidate blanket, and *shrinkage*, where we remove potential false positives from the CMB.
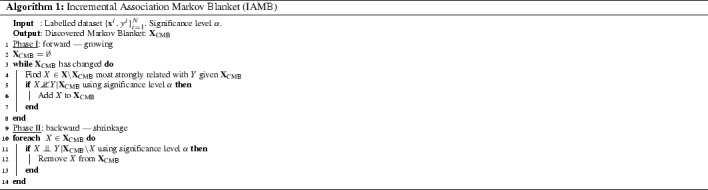


Importantly, lines 5 and 11 in this algorithm involve hypothesis tests between feature node *X* and label node *Y*. In our research, we are interested in how these tests would be if *Y* is a *semi-supervised* node in the network. In the literature, there is currently only one *inference-free* algorithm to derive the MB in this situation, the BAyesian Semi-SUpervised Method (BASSUM), by Cai et al. (2011), which turns out to have some limitations, discussed later in this section.

### Feature selection by ranking—information theoretic methods

Feature selection using mutual information is an extremely popular approach—Brown et al. (2012) surveyed over a dozen selection criteria published in various bodies of literature. In these approaches, we *rank* the features according to a score measure, and select the ones that have a higher score. For example, by ranking the features according to their estimated mutual information with the target, we derive a ranking that takes into account the *relevancy* to the class label. The score for feature $X_k$ is:

1 $$\begin{aligned} J_{MIM}(X_k)=\hat{I}(X_k;Y). \end{aligned}$$

This does not, however, take into account the *redundancy* between the features. By using more advanced techniques, we can take into account both the relevancy and the redundancy between the features themselves. For example, a popular criterion is mRMR (Peng et al. 2005), which ranks the features according to the score:

2 $$\begin{aligned} J_{mRMR}(X_k)=\hat{I}(X_k;Y) - \frac{1}{|{\mathbf{X}}_{\theta }|}\sum _{X_j \in {\mathbf{X}}_{\theta }}\hat{I}(X_k;X_j), \end{aligned}$$

where ${\mathbf{X}}_{\theta }$ is the set of the features already selected. Whilst this is popular, in an extensive empirical study Brown et al. (2012) found it to be quite unstable, in that the chosen set of features can vary wildly with small variations in the training data. Instead, they suggest the use the *Joint Mutual Information* (JMI) criterion (Yang and Moody 1999), where the score is conditional on the set already chosen:

3 $$\begin{aligned} J_{JMI}(X_k) = \sum _{X_j \in {\mathbf{X}}_{\theta }}\hat{I}(X_k;Y|X_j). \end{aligned}$$

The framework of Brown et al. (2012) focused only on fully-supervised data—our work, by using surrogate variables in an informed way, naturally extends this to semi-supervised scenarios.

In the semi-supervised literature there is one recent work on information theoretic methods, by He et al. (2016)—their proposal is MINT, a semi-supervised method based on mRMR. The main limitation of MINT is that it makes the traditional semi-supervised assumption, that the labelled set is an *unbiased* sample. To aid in understanding this further, in the next section we survey the various possible sampling assumptions in the semi-supervised literature.

### Semi-supervised learning

A semi-supervised dataset $\mathcal {D} = \{\mathcal {D}_L \cup \mathcal {D}_U\}$ can be seen as a combination of two samples: the labelled $\mathcal {D}_L$ and the unlabelled $\mathcal {D}_U$. We will assume that we have *N* examples, out of which $N_L$ belong to the labelled set, while $N_U$ to the unlabelled. For the labelled set we have the class labels $\mathcal {D}_L = \{ {\mathbf{x}}^i, y^i \}_{i=1}^{N_L}$, while for the unlabelled set we record only the feature vector $\mathcal {D}_U = \{ {\mathbf{x}}^{N_L+i}\}_{i=1}^{N_U}$.

In our analysis we will follow a data scenario known as “*single-training set*” (Elkan and Noto 2008). Here we assume that firstly we sample the whole dataset $\mathcal {D}$, and then we label some examples to form the labelled set $\mathcal {D}_L$, and the remaining examples form the unlabelled set $\mathcal {D}_U$. A convenient way to analyse this scenario is to introduce a binary random variable *S* in order to describe if an example is labelled, where $s=1$ or unlabelled, where $s=0$. The training data $\mathcal {D}$ are imagined to be drawn from $p({\mathbf{X}},Y,S)$—for each observation $\{ {\mathbf{x}},y,s\}$, the values of $\{{\mathbf{x}},s\}$ are recorded. But, we only record the value of *y* when $s=1$, otherwise it is labelled as “missing”. So what we actually can observe is not *Y*, but a “surrogate” variable $\widetilde{Y},$ taking on the true label value, *y*, when $s=1$, and a token “?” when $s=0$. In this way, the labelled set $\mathcal {D}_L$ comes from the joint distribution $p({\mathbf{x}},y|s=1)$, while the unlabelled set $\mathcal {D}_U$ from the distribution $p({\mathbf{x}}|s=0)$.

**Fig. 2:**
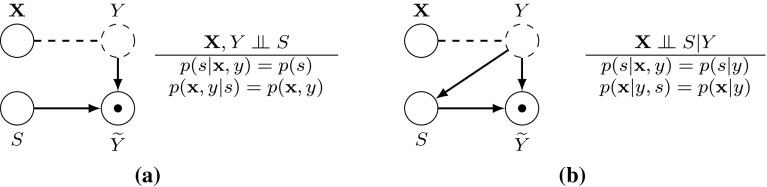
Graphical representation of the different semi-supervised scenarios: **a** the missingness mechanism *S* does not depend directly on features ${\mathbf{X}}$ or on class *Y* (MCAR: Traditional semi-supervised) and **b** the missingness mechanism *S* depends directly on the class *Y*. *Solid nodes* represent fully observed variables, while a *dashed node* is partially observed (i.e. with missing values) (MAR-C: Class-prior-change semi-supervised). Nodes with a dot  are fully observed variables $\widetilde{Y}$—taking the value of *Y* for the labelled examples and the token “?” for unlabelled examples—we refer to this as a *surrogate*. The *dashed line* between *X* and *Y* indicates there may or may not be a correlation between two variables. **a** MCAR: Traditional semi-supervised. **b** MAR-C: Class-prior-change semi-supervised

The key variable here then, is *S*, and the underlying (hidden) mechanism deciding whether a data sample is labelled, or not. This underlying mechanism could take several forms, and the exact form turns out to be very important for feature selection. To represent this, we adopt the formalism of *m*-graphs (Mohan et al. 2013), shown in Fig. 2.

#### Traditional semi-supervised scenario (or MCAR)

In Fig. 2a, we see the MCAR assumption (Little and Rubin 2002). Here, the *S* node is disconnected from both *X* and *Y*, so data samples are labelled purely at random. As a result, we have no selection bias in the labelled set, which turns out to be a useful property. According to Smith and Elkan (2007) this is the assumption that most semi-supervised learning methods use, including the very earliest work (Seeger 2002).

#### Class-prior-change semi-supervised scenario (or MAR-C)

In Fig. 2b, we see the MAR-C assumption, where *S* is a function of the true (unobservable) class label. In the missing data literature (Little and Rubin 2002), this scenario is classified as *missing-not-at-random*, and, since the missingness depends only in the class, Moreno-Torres et al. (2012) name it as *missing-completely-at-random class dependent (MAR-C)*. We can connect the two semi-supervised scenarios further, with the following observation: MCAR can be seen as a special case of MAR-C. When MAR-C holds we have $p(s=1|{\mathbf{x}},y) = p(s=1|y)$, while we can derive MCAR if we furthermore assume $p(s=1|y) = p(s)$ for each ${\mathbf{x}} \in {\mathbf{\mathcal {X}}}$ and $y \in \mathcal {Y}$.

The MAR-C scenario is important to understand, as it corresponds to a very practical semi-supervised situation, where there exists some bias in the labelling—certain classes of object may be more likely to receive a label than others. In the following paragraphs, we discuss this and the related literature.

Plessis and Sugiyama (2012) defined the scenario of *class-prior-change*, which occurs when the class balance in the labelled set does not reflect the population: $p(y=1|s=1) \ne p(y=1)$. For example, as discussed in the introduction, this might occur in survey data when there is some stigma associated with reporting the true value of *Y*. A more restricted version of this, where we observe examples only from the positive class, generates *positive-unlabelled* data under the widely used *selected completely at random* assumption (Elkan and Noto 2008). In this case, there is conditional independence at the *event level*: , while there are no negatively labelled examples $p(s=1|y=0)=0$. A common approach to solve this problem is to simply assume unlabelled examples are negatives. This approach has been shown to be powerful in the literature of positive-unlabelled learning (Elkan and Noto 2008, Sect. 2) and in semi-supervised novelty detection (Blanchard et al. 2010, Sect. 3). Exploring how this simple strategy performs in terms of feature selection is the main focus of our work.

Furthermore, it is interesting to mention that in the MAR-C scenario it is not possible to consistently estimate *p*(*y*) directly from the observed data, without further modelling assumptions. However, if we have some prior knowledge of *p*(*y*), the bias introduced by this sampling mechanism can be corrected (Hein 2009). Our work explores how we can use this prior knowledge to decide which is the optimal simple strategy for semi-supervised feature selection.

### Motivating an inference-free approach and related work

In the literature there are two main methods for handling missing data (Allison 2001): (1) inference-free methods (a.k.a. model-independent) such as listwise deletion or dummy variable adjustment methods, and (2) inference-based methods (a.k.a. model-dependent) such as expectation maximization or single/multiple imputation.

For the task of feature selection, in order to be consistent with the *filter* principle (Guyon et al. 2006), we need to follow a model-independent approach. Two simple assumptions with nice theoretical properties we can make are to assume the unlabelled examples positive, or assume them negative. With our work we will explore the consequences of these assumptions in feature selection through hypothesis testing (Sect. 3) and ranking (Sect. 4).

Apart from being consistent with the filter assumption, *inference-free* approaches have other important advantages. Van den Broeck et al. (2015) present some of them in the context of estimating the parameters of a Bayesian network. The most important is that inference-free methods are efficient: expressed in closed-form, requiring only a single pass over the data. This is a significant computational advantage over inference-based methods.

Cai et al. (2011) suggest an inference-free algorithm for semi-supervised feature selection using a modified test of independence. This algorithm implicitly makes the traditional semi-supervised assumption that the labelled set is an unbiased sample from the overall population—it makes use of the unlabelled examples to improve the reliability of conditional independence tests. For example, to estimate the *G*-statistic, Eq. (7) in Appendix A, it uses both labelled and unlabelled data for the observed counts $o_{.,.,{\mathbf{z}}}$ and $o_{x,.,{\mathbf{z}}}$. This technique is known in statistics as *available case analysis* or *pairwise deletion*. The problem with pairwise deletion is the resultant ambiguity over the definition of the overall sample size, which is crucial for deriving standard errors and sampling distributions; the interested reader can find more details in Allison (2001, page 8). This can lead to unpredictable results, for example there are no guarantees that the *G*-statistic will follow a $\chi ^2$-distribution after this substitution. Another weakness of BASSUM is that it cannot be applied in restricted semi-supervised environments where we have labelled examples *only* from one class, which is the case for positive-unlabelled learning. Our work (Sect. 3) suggests ways for deriving the MB around of *any kind* of semi-supervised node (i.e. semi-supervised with class-prior-change, or positive-unlabelled). The main idea is to explore the consequences of testing conditional independence (i.e. Algorithm 1—Lines 5 and 11) by using the surrogate approaches: assume the unlabelled data are all positives or assume are all negatives. The result of our analysis is a new algorithm, *Semi*-IAMB (Algorithm 2), and Sect. 5 presents applications on how it performs in different semi-supervised scenarios.

Finally, He et al. (2016) suggest MINT, an extension of a popular information theoretic feature ranking criterion, the mRMR criterion (Peng et al. 2005), for inference-free semi-supervised feature selection. MINT uses only the labelled set to estimate the relevancy, i.e. the first term on the *RHS* of Eq. (2), and both labelled and unlabelled examples for estimating the redundancy, i.e. the second term. The main limitation of this approach is that it makes the MCAR assumption. Our work (Sect. 4) suggests a generic method for extending any feature ranking criterion suggested in fully-labelled scenarios (Brown et al. 2012) to semi-supervised scenarios. Our analysis is based on using surrogate fully observable variables in place of the semi-supervised target, and explore how these surrogates perform on MCAR and MAR-C semi-supervised scenarios. The result of our analysis is a new algorithm, *Semi*-JMI (Algorithm 3), and Sect. 6 presents the empirical performance of this approach in several semi-supervised datasets.

## Hypothesis testing in semi-supervised scenarios

In this section we examine the theoretical/empirical properties of semi-supervised hypothesis testing with surrogate variables.

### Surrogate approaches for hypothesis testing

As discussed in the previous sections, the two surrogates we study are ${\widetilde{Y}_0}$, where we assume all unlabelled examples are negative, and $\widetilde{Y}_1$, where we assume all unlabelled examples are positive. In addition, we examine a “default” option—to just *ignore* the unlabelled examples and use only the labelled set $\mathcal {D}_L$. This is the baseline for any semi-supervised learning, and it is equivalent to standard supervised learning *but* with smaller sample size. To the best of our knowledge, our work is the first that explores the theoretical properties and consequences of ignoring unlabelled data, for information theoretic feature selection. Thus, in the rest of our analysis we will theoretically and empirically analyse the following three approaches:

**Surrogate 1** $$(\mathcal {D}_L)$$:
Ignore unlabelled examples, i.e. use only $\mathcal {D}_L$.
**Surrogate 2** $$({\widetilde{Y}_0})$$:
Assume unlabelled examples are negative, i.e. use surrogate ${\widetilde{Y}_0}$.
**Surrogate 3** $$(\widetilde{Y}_1)$$:
Assume unlabelled examples are positive, i.e. use surrogate $\widetilde{Y}_1$.

As we saw in Sect. 2.1, the test of independence plays a crucial role in feature selection through MB discovery. Therefore it is important to analyse theoretically the consequences of testing independence by using surrogate approaches instead of the unobservable fully-supervised target variable *Y*. The two factors that characterise a hypothesis test are: the *false positive* rate (FPR or type-I error), and the *false negative* rate (FNR or type-II error). The FPR is the probability of falsely stating a dependency exists $X-Y$, when in fact there is none. The FNR is the opposite, falsely stating there is no dependency. If a surrogate test has the same FPR and FNR as the ideal test, then (in the context of feature selection) the *exact same* features will be selected, despite the missing labels. The challenge is therefore to prove what the FPR/FNR will be for each surrogate, with different sampling assumptions (more details about these two types of error can be found on the tutorial of hypothesis testing in Appendix A).

In IAMB (Algorithm 1), the probability of a false positive ($\alpha$) is a user specified input parameter, thus, we need to identify surrogates that are consistent with this user specification. If we have more than two approaches that are consistent, the more desirable will be that which performs better in terms of the FNR. Now we will formally define these two desirable properties.

Given a fully observed feature *X*, a partially observed *Y*, and a fully observed surrogate $\widetilde{Y}$, we define two properties that $\widetilde{Y}$ may possess, *validity* and *informedness*, concerning the false positive and the false negative rate of the test of independence when $\widetilde{Y}$ is used instead of *Y*.

#### Definition 1

(*Valid surrogate*) The surrogate $\widetilde{Y}$ is *valid* for a hypothesis test if, and only if, it has the same FPR as the ideal test using *Y*:If *X* is independent of $\widetilde{Y}$, then *X* is also independent of *Y*, and vice versa.

#### Definition 2

(*Informed surrogate*) The surrogate $\widetilde{Y}$ is *informed* for a hypothesis test if, and only if, the following conditions hold:

1. it has the same FPR, i.e. is a *valid* surrogate (see Definition 1)
2. the test can be corrected to have the same FNR as the ideal test, by increasing the number of samples by a factor $\kappa$, calculated from user-supplied knowledge of the class probability.

Thus, when a surrogate is informed, we can use it for hypothesis testing as a perfect surrogate for *Y*, since we know that (if we can find sufficient extra samples) the FPR and FNR will be identical to that of the ideal (unobservable) test. In the following, we will present results of theory work proving the *validity*/*informedness* of various semi-supervised hypothesis testing and ranking scenarios.

### Testing: labels missing completely at random (MCAR)

In order to use any of the three surrogate approaches we should first explore if they are *valid* for testing the null hypothesis of independence. Or in other words, we ensure that following a surrogate approach, the probability of a type I error ($\alpha$) will be the same as if we had used the (unobservable) fully-supervised test between *X* and *Y*. The following theorem presents our first findings.

#### Theorem 1

(MCAR: Which surrogate tests are *valid* for testing ?) Under the MCAR assumption, all three surrogates are valid:


#### Proof

Sketches for each of these situations can be found in Appendix B.1. $\square$

While Theorem 1 tells us that the surrogate tests are equivalent to the unobservable test for detecting independencies, it says nothing about how well the surrogate approaches perform when the null hypothesis is *false*. In that case we should compare the tests in terms of their power to detect a given effect. The effect size that our work uses is the mutual information—*I*(*X*; *Y*)—which quantifies the dependency between the random variables, and it is the natural effect for the *G*-test of independence (Appendix A).

We will now explore the power of *surrogate* *G*-tests of independence in order to detect effects expressed in terms of *I*(*X*; *Y*). To do so we will re-express the non-centrality parameters of the surrogate tests in terms of the non-centrality parameter of the unobservable test—$\lambda _{G(X;Y)}=2NI(X;Y).$ With the following theorem we show that the non-centrality parameters of the surrogate tests can be written in terms of the non-centrality parameter of the unobservable test as $\lambda _{G(X;\widetilde{Y})} = \kappa \lambda _{G(X;Y)},$ and we derive the three $\kappa$ correction factors.

#### Theorem 2

(MCAR: Informed surrogate approaches) Under the MCAR assumption, all three surrogate are informed, with correction factors:

$$\begin{aligned}&\mathbf{Surrogate~1}~(\mathcal {D}_L):~\kappa _1 =p(s=1),&\\&\mathbf{Surrogate~2}~({\widetilde{Y}_0}):~~\kappa _2 = \frac{1-p(y=1)}{1-p(y=1)p(s=1)} p(s=1),&\\&\mathbf{Surrogate~3}~(\widetilde{Y}_1):~~\kappa _3 = \frac{1-p(y=0)}{1-p(y=0)p(s=1)} p(s=1).&\end{aligned}$$

#### Proof

Can be found in Appendix B.2. $\square$

Since all three correction factors are smaller than one, we conclude that all three surrogate tests have smaller non-centrality parameters than the fully-supervised test, and as a result smaller power. The loss in power, which is captured by the correction factors, depends on $p(y=1)$ and $p(s=1)$. We can have an unbiased estimate for the first probability from the labelled set, while, by the number of labelled examples, we can calculate and control the second probability. Furthermore we have that: $\lambda _{G(X;Y|s=1)} > \lambda _{G(X;{\widetilde{Y}_0})}$ and $\lambda _{G(X;Y|s=1)} > \lambda _{G(X;\widetilde{Y}_1)}$, and since all of these three tests have the same degrees of freedom we can derive the following corollary.

#### Corollary 1

(MCAR: Comparing the power of the surrogate tests) In MCAR the most statistically powerful of the three surrogate approaches is **Surrogate 1**, that is, to simply ignore the unlabelled data.

**Fig. 3:**
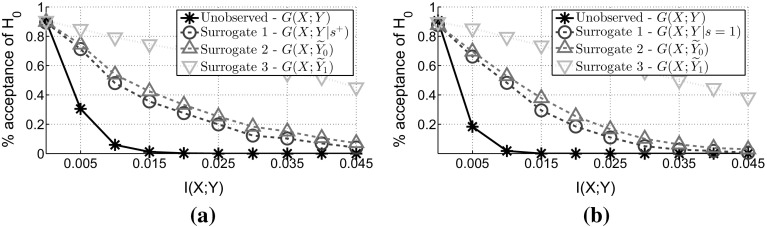
**MCAR**: Comparing the False Positive and False Negative rate. For all figures we have $\alpha = 0.10$, while to generate the semi-supervised dataset we used $p(s=1)=0.25$. **a** $N=500$ and $|\mathcal {X}|=2$. **b** $N=1000$ and $|\mathcal {X}|=5$

To add experimental support to our theoretical results, we generate synthetic random variables *X* and *Y* with different degrees of dependency and we plot figures similar to the figures in Gretton and Györfi (2010). To create the data, firstly we generate the values of *Y*, by taking *N* samples from a Bernoulli distribution with $p(y=1)$. Then, we randomly choose the parameters *p*(*x*|*y*) that guarantee the desired degree of dependency expressed in terms of *I*(*X*; *Y*) and we use these parameters to sample the values of *X*. In the *x*-axis of the figures we have different effect sizes in terms of the mutual information between *X* and *Y*,  while in the *y*-axis we have the acceptance rate of the null hypothesis $H_0$ (over 1000 independent generations of the data). The *y*-intercept represents *1—False Positive Rate*, and should be close to $1-\alpha$ in order for the tests to be valid, while elsewhere the plots indicate the *False Negative Rate*. Figure 3 verifies Theorem 1, by showing that the surrogate tests are valid, since all lines have the same intercept at $1-\alpha = 0.90$ and as a result the surrogate tests have the same false positive rate. Furthermore, all of the surrogate approaches lead to tests with higher false negative rate—this verifies Theorem 2 that the three tests have less power than the unobservable test. Finally, from the same figure we observe that the most powerful surrogate approach is to ignore the unlabelled examples, which verifies Corollary 1.

### Testing: labels missing at random class dependent (MAR-C)

When the labels are MAR-C we prove with the following theorem that we have the same valid tests as in the MCAR scenario (Sect. 3.2).

#### Theorem 3

(MAR-C: Which surrogate tests are *valid* for testing ?) Under the MAR-C assumption, all three surrogates are valid.


#### Proof

Sketches for each of these situations can be found in Appendix B.3. $\square$

All three surrogates are valid—in the following we determine whether they are also *informed*. To do this, we re-express the non-centrality parameters of the two valid surrogate tests, $G(X;{\widetilde{Y}_0})$ and $G(X;\widetilde{Y}_1)$, in terms of the non centrality parameter of the unobservable fully-supervised test, *G*(*X*; *Y*).

#### Theorem 4

(MAR-C: Informed surrogate approaches) In MAR-C only two of the surrogates—**Surrogate 2** and **Surrogate 3**—are also informed, with the following correction factors:

$$\begin{aligned} \mathbf{Surrogate~2}~({\widetilde{Y}_0}): ~~ \kappa _{{\widetilde{Y}_0}} = \frac{1-p(y=1)}{p(y=1)} \frac{p({\widetilde{y}_0=1})}{1-p({\widetilde{y}_0=1})},&\\ \mathbf{Surrogate~3}~(\widetilde{Y}_1): ~~ \kappa _{\widetilde{Y}_1} = \frac{1-p(y=0)}{p(y=0)} \frac{p(\widetilde{y}_1=0)}{1-p(\widetilde{y}_1=0)}.&\end{aligned}$$

#### Proof

Can be found in Appendix B.4. $\square$

The probabilities $p({\widetilde{y}_0=1})$ and $p(\widetilde{y}_1=0)$ can be calculated and controlled through the examples that are *labelled* as positives and negatives. But, as we mentioned in Sect. 2.3, when the labels are MAR-C we cannot use the labelled set to consistently estimate the probability $p(y=1)$. From the above theorem, we observe that by using *“exact” prior knowledge* over $p(y=1)$, we can quantify the power of these two surrogate approaches. As a result we can use the $G(X;{\widetilde{Y}_0})$ and/or $G(X;\widetilde{Y}_1)$ instead of the *G*(*X*; *Y*) for power analysis and sample size determination. Taking advantage of the extra degree of freedom in $p({\widetilde{y}_0=1})$ and/or $p(\widetilde{y}_1=0)$, we can also determine the *required level of supervision* (i.e. number of labelled examples) needed, following the same procedure as in sample size determination. In our previous work (Sechidis et al. 2014), we presented a complete methodology for sample/labelled size determination in positive-unlabelled scenarios by using the $\kappa _{{\widetilde{Y}_0}}$ correction factor and surrogate ${\widetilde{Y}_0}$.

Interestingly, to decide which of these two tests is more powerful we do not need exact prior knowledge, but we can do so by using some *“soft” prior knowledge* expressed in terms of inequality. Before presenting this result, let us define first the following threshold:

#### Definition 3

(*Switching threshold*) The switching threshold value ($\phi$) is

$$\begin{aligned} \phi = \frac{1}{1+\sqrt{\frac{(1-p({\widetilde{y}_0=1}))p(\widetilde{y}_1=0)}{p({\widetilde{y}_0=1})(1-p(\widetilde{y}_1=0))}}}. \end{aligned}$$

This threshold can be estimated using the values of the observed variables ${\widetilde{Y}_0}$ and $\widetilde{Y}_1$. Let *p* be the number of positively labelled examples, *n* be the number of negatively labelled examples, and *m* the number of unlabelled examples. Then a consistent estimator of $p({\widetilde{y}_0=1})$ is $p/(p+n+m)$, while $p(\widetilde{y}_1=0)$ as $n/(p+n+m)$. With some straightforward algebra the estimated threshold can be written as: $a/(a+b)$, where and $a=\sqrt{p(p+m)}$, and $b=\sqrt{n(n+m)}$.

Using this threshold and some user-specified “soft” prior knowledge over $p(y=1)$ we can decide the most powerful option by the following theorem.

#### Theorem 5

(MAR-C: Comparing the power of the two surrogate tests) Under the MAR-C assumption, if the following inequality holds, the most statistically powerful option (i.e. lowest false negative rate) is **Surrogate 2**.

4 $$\begin{aligned} p(y=1) < \phi \end{aligned}$$

When the opposing inequality holds, the most powerful choice is **Surrogate 3**. When equality holds, both approaches are equivalent.

The proof of this theorem is straightforward. $G(X;{\widetilde{Y}_0})$ is more powerful than $G(X;\widetilde{Y}_1)$ when $\kappa _{{\widetilde{Y}_0}} > \kappa _{\widetilde{Y}_1}$, which results in the inequality $p(y=1) < \phi$. When the opposing inequality holds, the most powerful choice is $G(X;\widetilde{Y}_1)$. When equality holds, both approaches are equivalent, since they have the same correction factors, and as a result the same non-centrality parameters.

Unfortunately a conclusion for the “ignore unlabelled” strategy seems intractable, since it involves expressing the non-centrality parameter, $\lambda _{G(X;Y|s=1)}$, in terms of the non-centrality parameter of the unobservable fully-supervised test, $\lambda _{G(X;Y)}$. Combining our findings on the MAR-C scenario with our findings on the MCAR scenario (Sect. 3.2), we can consider the following conjecture:

#### Conjecture 1

(MAR-C: Comparing the power of the tests) The closer we are to the MCAR, i.e. $D_{KL}(p(y)||p(y|s=1)) \approx 0$, then **Surrogate 1**, $G(X;Y|s=1)$, will have the highest statistical power. In contrast, the closer we are to extreme MAR-C scenarios, i.e. $D_{KL}(p(y)||p(y|s=1)) \gg 0$, then either **Surrogate 2** or **3**, that is $G(X;{\widetilde{Y}_0})$ or $G(X;\widetilde{Y}_1)$, will have the highest power. In this latter scenario we can identify which of the two will be most powerful using Theorem 5.

A theoretical justification for this conjecture, requires all three surrogate tests to be informed in both MCAR and MAR-C scenarios. As we mentioned this seems to be intractable, since in MAR-C, is highly non-trivial exercise to derive a closed form relationship between $\lambda _{G(X;Y|s=1)}$ and $\lambda _{G(X;Y)}$. An intuitive justification can come from our observation in Sect. 2.3 that MCAR is as a restricted version of MAR-C. Now we will provide empirical evidence.

**Fig. 4:**
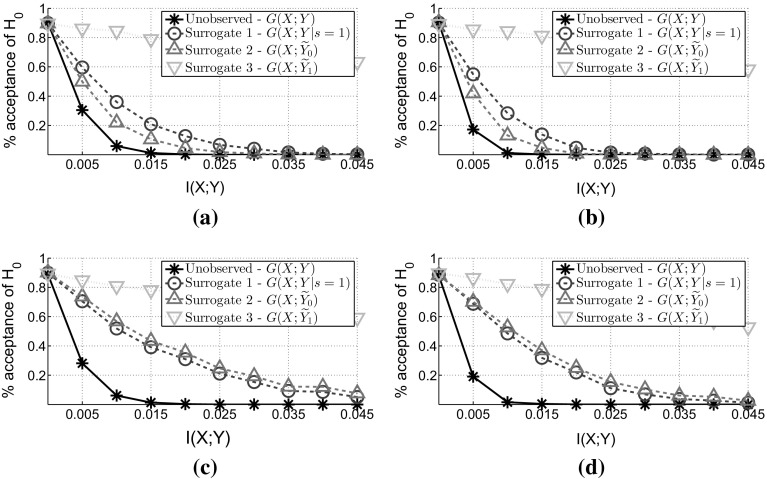
**MAR-C**: Comparing the False Positive and False Negative rate. For all figures we have $\alpha = 0.10$ and $p(y=1)=0.20$. In the *first row*, figures (**a**) and (**b**), we label the data such that $p(y=1|s=1) =0.50$—an extreme MAR-C scenario since $D_{KL}(p(y)||p(y|s=1))=0.19$. While in the second row, figures (**c**) and (**d**), we label the data such that $p(y=1|s=1) = 0.25$—a MAR-C scenario close to MCAR since $D_{KL}(p(y)||p(y|s=1))=0.01$. **a** $|\mathcal {X}|=2$, $N=500$ and $p(s=1)=0.25$. **b** $|\mathcal {X}|=5$, $N=1000$ and $p(s=1)=0.25$. **c** $|\mathcal {X}|=2$, $N=500$ and $p(s=1)=0.20$. **d** $|\mathcal {X}|=5$, $N=1000$ and $p(s=1)=0.20$

Figure 4 verifies Theorem 3 by showing that any of the three surrogate tests is a valid approach, since all of the lines have the same intercept (at $1-\alpha$) and as a result the tests have the same false positive rate. Furthermore, we can verify Theorem 5 by incorporating “soft” prior knowledge over $p(y=1)$ and using inequality (4) to decide which of the two tests, $G(X;{\widetilde{Y}_0})$ or $G(X;\widetilde{Y}_1)$, is more powerful. For the first setting (Fig. 4a, b) we have $p({\widetilde{y}_0=1}) = p(\widetilde{y}_1=0) = 0.125$, so the *RHS* of inequality (4) is equal to 0.50. And by using “soft” knowledge that $p(y=1)$ is less than this value we can conclude that $G(X;{\widetilde{Y}_0})$ is more powerful than $G(X;\widetilde{Y}_1)$ and Fig. 4a, b verify this conclusion. The same also holds for the second setting (Fig. 4c, d) where we have $p({\widetilde{y}_0=1}) = 0.05$ and $p(\widetilde{y}_1=0) = 0.15$ and the *RHS* of the inequality (4) becomes 0.35. Again, by using “soft” knowledge over $p(y=1)$, we can conclude that $G(X;{\widetilde{Y}_0})$ is more powerful than $G(X;\widetilde{Y}_1)$.

By comparing the first setting (first row Fig. 4a, b) with the second setting (second row Fig. 4c, d), we can verify Conjecture 1. In the first setting, the MAR-C is more extreme. So, in this scenario, using the unlabelled examples assuming that they belong to the negative class outperforms the other approaches. While in the second setting we are closer to the MCAR assumption. As a result, in this scenario we can see that ignoring the unlabelled examples is more powerful option.

An interesting point to mention is that our analysis in this section can be also used when we have labelled examples from one class, such us the positive-unlabelled setting. Under the positive-unlabelled constraint the surrogate variable of assuming all unlabelled examples being negative (${\widetilde{Y}_0}$) is valid and it is also informed by incorporating prior knowledge over $p(y=1)$. As a result we can use the $G(X;{\widetilde{Y}_0})$-test for experimental design activities, such as sample size determination. This application of our work was presented in Sechidis et al. (2014), where we also explored how we to incorporate uncertain prior knowledge over $p(y=1)$.

### Conditional independence tests in semi-supervised learning

The results that we proved for testing in MCAR (Sect. 3.2) and MAR-C (Sect. 3.3) can be extended to conditional tests. The MCAR extension is straightforward, because of the unconditional independence presented in Fig. 2a. Deriving the results in MAR-C is more challenging and this is the focus of the current section. Firstly we will show that testing conditional independence by assuming the unlabelled examples to be either positive or negative is a valid approach.

#### Theorem 6

(MAR-C: Which surrogate tests are valid for testing  ?) In MAR-C we can test conditional independence by these three surrogate approaches:


#### Proof

Sketches can be found in Appendix B.5. $\square$

The consequence of this theorem is that the derived conditional tests of independence are valid, but it does not tell us anything about what is happening when the alternative hypothesis holds. To explore that, we will quantify the amount of power that we are losing by assuming all unlabelled examples are negative (i.e. using ${\widetilde{Y}_0}$) or positive (i.e. using $\widetilde{Y}_1$).

#### Theorem 7

(MAR-C: Informed surrogates for the conditional test) In MAR-C only two valid tests—**Surrogate 2** and **3**—are also informed with the same correction factors as the ones for the unconditional tests presented in Theorem 4.

#### Proof

Can be found in Appendix B.6. $\square$

The correction factors in the non-centrality parameters of the unconditional tests (Theorem 4) are the same as the ones of the conditional tests (Theorem 7), thus we can use inequality (4) and incorporate *“soft” prior knowledge* to decide which surrogate approach is most powerful. Section 5.1 presents an experimental verification of the correctness of these factors in the context of MB discovery, and we show how we can use them to derive the MB of positive-unlabelled and semi-supervised target nodes using “exact” and “soft” prior knowledge.

### The switching procedure applied to Markov Blanket discovery—*Semi*-IAMB

We now define an algorithm based on the observations of this section, *Semi*-IAMB (Algorithm 2). While IAMB decides the optimal feature set around fully-supervised targets *Y* by testing conditional independence, in the semi-supervised scenarios we can use Theorem 7 and “soft” prior knowledge to decide the most powerful surrogate choice between ${\widetilde{Y}_0}$ and $\widetilde{Y}_1$. If inequality (4) holds we should choose ${\widetilde{Y}_0}$ instead of $\widetilde{Y}_1,$ and when the opposing inequality holds the most powerful choice is $\widetilde{Y}_1$—when equality holds, both approaches are equivalent. After deciding which is the most powerful option, we use IAMB (Algorithm 1) with this surrogate variable.
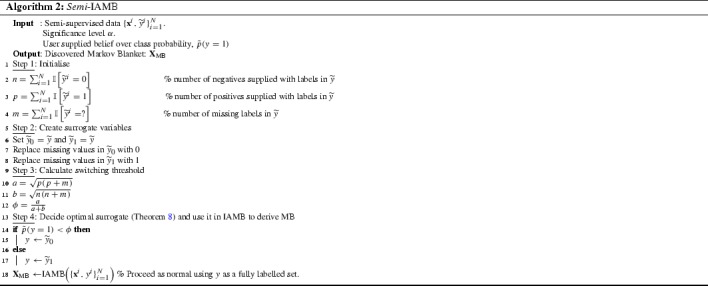


Section 5 compares the performance of *Semi*-IAMB against other semi-supervised approaches. Before that, in the following section, we will show how to use surrogates to derive feature *rankings* in semi-supervised scenarios.

## Ranking features in semi-supervised scenarios

In the previous section we studied *hypothesis testing* in semi-supervised data—in some situations we may not be so interested in a yes/no hypothesis test, but instead in a full *ranking* of all features, in relation to the label.

### Surrogate approaches for feature ranking

The main question we answer in this section is to decide which surrogate variable to use in order to rank the features, as close as possible to the population ranking that would be achieved if we could use the (unobservable) target *Y*. To do so we will build on the results in the previous section, and suggest efficient ways for feature ranking under the two different missingness scenarios. Before that we should give a formal definition on the equivalence between rankings derived using different approaches.

#### Definition 4

(*Ranking equivalence*) Assume that we have a set of features ${\mathbf{X}} = \{ X_1,\ldots ,X_d\}$ and we use two different approaches to rank them, e.g. $J^Y$ which uses the mutual information between the features and the unobservable variable *Y*, and $J^{\widetilde{Y}}$ which uses the mutual information between the features and the surrogate ${\widetilde{Y}}$. We say that the two approaches $J^Y$ and $J^{\widetilde{Y}}$ are *ranking equivalent*, $J^{Y} \mathop = \limits ^R J^{{\widetilde{Y}}}$, if $\forall ~i,~j$ it holds that:

$$\begin{aligned} J^Y(X_i)< J^Y(X_j) \Leftrightarrow J^{\widetilde{Y}}(X_i) < J^{\widetilde{Y}}(X_j), \end{aligned}$$

where $J^{Y}(X_i)$ and $J^{\widetilde{Y}}(X_i)$ represent the score of the feature $X_i$ estimated by the two different approaches.

For example, assume that we have a set of features ${\mathbf{X}}$, two random variables *Y* and ${\widetilde{Y}}$, and we use $J^Y=I(X_i;Y)$ the mutual information (MIM) scoring criterion. If it were to hold that $I(X_i;Y) = \kappa I(X_i;{\widetilde{Y}})~\forall ~X_i~\in ~{\mathbf{X}}$, with $\kappa \in \mathbb {R}^+$ is constant with respect to the *X*’s, then we know they are ranking equivalent: $J_{MIM}^Y \mathop = \limits ^R J_{MIM}^{\widetilde{Y}}$.

### Ranking: labels missing completely at random (MCAR)

With straightforward algebra (see proof of Theorem 2) we can derive the following:

$$\begin{aligned}&\mathbf{Surrogate~1}~(\mathcal {D}_L):~I(X;Y|s=1) = I(X;Y),&\\&\mathbf{Surrogate~2}~({\widetilde{Y}_0}):~~I_2(X;{\widetilde{Y}_0}) = \frac{p(s=1)-p(s=1)p(y=1)}{1-p(y=1)p(s=1)}I_2(X;Y),&\\&\mathbf{Surrogate~3}~(\widetilde{Y}_1):~~I_2(X;\widetilde{Y}_1) = \frac{p(s=1)-p(s=1)p(y=0)}{1-p(y=0)p(s=1)}I_2(X;Y).&\end{aligned}$$

where $I_2(X;Y)$ is the squared-loss mutual information (Sugiyama 2012), which is asymptotically equivalent to *I*(*X*; *Y*) (more details in Appendix A).

We see that all of the mutual information quantities of the *LHS* can be written as $\kappa$ times the mutual information derived by using the unobservable variable *Y*,  where the factor $\kappa$ is independent of the characteristics of the feature *X*. So a direct consequence of these relationships is that *we can use the surrogate approaches to rank the features, and the ranking will be the same as if we had used the unobservable target* *Y*.

Deciding which of the above approximate rankings is preferable in finite sample has to do with the accuracy of the estimators. There is a natural relationship between testing and estimation, and as Loftus (1991) mentions “The more power you have, the smaller are your confidence intervals, i.e., the better your knowledge of where population means are”. So by exploring the power of tests, we can derive estimators with higher accuracy, which will result to rankings that are closer to the population one. In Sect. 3.2, we showed that the most powerful option to test independence is to ignore the unlabelled examples, and thus this surrogate will result to a ranking that is closer to the population one. The above results are summarised in the following corollary.

#### Corollary 2

(MCAR: Ranking) In MCAR the rankings derived by the all three surrogates are ranking equivalent to the population ranking: $J_{MIM}^Y \mathop = \limits ^R J_{MIM}^{{\mathcal {D}}_L}$, $J_{MIM}^Y \mathop = \limits ^R J_{MIM}^{{\widetilde{Y}_0}}$ and $J_{MIM}^Y \mathop = \limits ^R J_{MIM}^{\widetilde{Y}_1}$. In finite datasets **Surrogate 1** is the optimal choice.

To verify Corollary 2 we will compare the rankings derived by using the different estimators against the population ranking. To check the similarity between the rankings we use Spearman’s $\rho$ correlation coefficient (Kalousis et al. 2007). The range of values that this coefficient takes is $[-1,1]$, where 1 means that the two rankings are identical, 0 means that there is no correlation between them. Since, to assess this, we need to have knowledge of the population ranking we will use a synthetic dataset—Table 1 presents the characteristics. This dataset is extremely challenging in terms of predicting the population ranking, because the stepwise increase in the population values of the mutual information is 0.0001. We sample various different dataset sizes (*N*) from 2500 (2.5*k*) to 500000 (500*k*) examples to observe the performance when the sample size increases.

**Table 1:** Characteristics of synthetic dataset used to observe the ranking performance

# Features	Population values of the effects between the features and the target	Class prob. $p(y=1)$
100	$I(X_1;Y) = 0.0351,~I(X_2;Y) = 0.0352,\ldots ,~I(X_{100};Y) = 0.0450$	0.20

The arity of features is chosen randomly between the following values $|\mathcal {X}|=2,5,10$ and 20

**Fig. 5:**
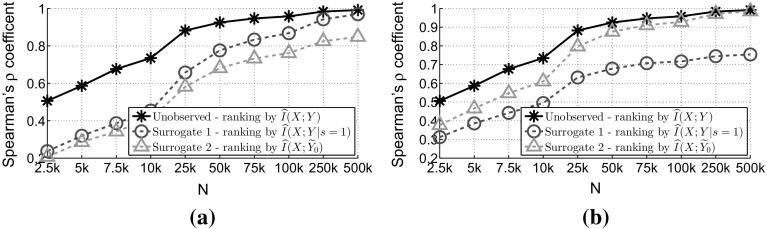
**“Extremely Challenging”** dataset—difference between relevant/irrelevant features is of the order $10^{-4}$ nats. Plot shows Spearman’s $\rho$ (average over 10 repetitions) between population ranking and ranking derived through different surrogate approaches. For each repetition we average over 30 semi-supervised versions with $p(s=1) = 0.25$ and sampled by: **a** MCAR and **b** MAR-C with $p(y=1|s=1) = 0.50$. To help the visibility, we plot only the most powerful option between surrogates 2 and 3, which in both scenarios is surrogate 2. **a** MCAR. **b** MAR-C

Figure 5a verifies the results of this section. Ignoring the unlabelled examples outperforms the other surrogate approaches. Furthermore we see that by increasing the sample size all of the estimators improve their rankings, and they are closer to the population ranking, this is a verification of the fact that *all* of the approaches converge to the population ranking.

### Ranking: labels are missing at random class dependent (MAR-C)

In this scenario it is impossible to derive relationships for **Surrogate 1** (i.e. ignore the unlabelled examples), but only for the following two surrogate approaches (see proof of Theorem 4):

$$\begin{aligned}&\mathbf{Surrogate~2}~({\widetilde{Y}_0}):~I_2(X;{\widetilde{Y}_0}) = \frac{1-p(y=1)}{p(y=1)} \frac{p({\widetilde{y}_0=1})}{1-p({\widetilde{y}_0=1})}I_2(X;Y),&\\&\mathbf{Surrogate~3}~(\widetilde{Y}_1):~I_2(X;\widetilde{Y}_1) = \frac{1-p(y=0)}{p(y=0)} \frac{p(\widetilde{y}_1=0)}{1-p(\widetilde{y}_1=0)}I_2(X;Y).&\end{aligned}$$

Again, we observe that all of the mutual information quantities of the *LHS* can be written as $\kappa$ times the mutual information derived by using the unobservable variable *Y*,  where the factor $\kappa$ is independent of the characteristics of the feature *X*. A consequence of these relationships is that the mutual information quantities of the *LHS* can be used to rank the features, and the ranking will be the same as if we had used the unobservable variable *Y*.

Another interesting consequence is that we can rank the features *without* an exact prior knowledge over the $p(y=1)$ by simply using Surrogate 2 or 3. Deciding the optimal choice between these two surrogates has to do with the accuracy of the estimators, which can be answered by using our findings in hypothesis testing (Sect. 3.3), since the most powerful test leads to more accurate estimators (Loftus 1991). Thus we can suggest the following theorem.

#### Theorem 8

(MAR-C: Ranking by using “soft” prior knowledge) In MAR-C the rankings derived by **Surrogate 2** or **Surrogate 3** are ranking equivalent to the population ranking: $J_{MIM}^Y \mathop = \limits ^R J_{MIM}^{{\widetilde{Y}_0}}$ and $J_{MIM}^Y \mathop = \limits ^R J_{MIM}^{\widetilde{Y}_1}$. Furthermore, when $p(y=1) < \phi$ holds the the optimal choice is **Surrogate 2** (${\widetilde{Y}_0}$) while when the opposing inequality holds, the most optimal choice is **Surrogate 3** ($\widetilde{Y}_1$). When equality holds, both approaches are equivalent.

Proving this theorem is straightforward, by using Theorem 5 and the fact that the most powerful way for testing will result in the most accurate estimator (Loftus 1991), and as a result a ranking that is closer to the ideal unobservable ranking. Figure 5b verifies the results of this section. To generate the semi-supervised data again we used the same methodology as in Sect. 3.3. When we have “soft” prior knowledge we can decide the optimal choice between $\hat{I}(X;{\widetilde{Y}_0})$ and $\hat{I}(X;\widetilde{Y}_1)$. In this setting, since $p({\widetilde{y}_0=1})=p(\widetilde{y}_1=0)=0.125$, the *RHS* of the inequality (4) becomes 0.50, which is larger than 0.20 and as result the ranking derived through $\hat{I}(X;{\widetilde{Y}_0})$ will be closer to the population ranking than the one derived by $\hat{I}(X;\widetilde{Y}_1)$.

### Extending to higher order criteria

Throughout this section we analysed rankings derived through MIM criterion, which at each feature selection step ranks the features by simple estimating $I(X_k;Y).$ More advanced criteria rank the features using higher-order conditional mutual information terms, i.e. JMI ranks the features by estimating $\sum _{X_j \in {\mathbf{X}}_{\theta }}I(X_k;Y|X_j).$ When the labels are MCAR or MAR-C, our results can be directly extended to these higher order rankings, because of independence and conditional independence assumptions presented in Fig. 2a, b, respectively. This can be formally proved by using same reasoning as in the proof of Theorem 7.

### The switching procedure applied to feature ranking—*Semi*-MIM, *Semi*-JMI

We now define two algorithms based on the observations of this section, *Semi*-MIM and *Semi*-JMI (Algorithm 3). Under our analysis we can use “soft” prior knowledge to decide which is the optimal surrogate to be used in order to rank the features. If inequality (4) holds we chose ${\widetilde{Y}_0}$ instead of $\widetilde{Y}_1$. When the opposing inequality holds the most powerful choice is $\widetilde{Y}_1$. When equality holds, both approaches are equivalent. After deciding which is the most powerful surrogate, we can use MIM or JMI criterion with this variable instead of the unobservable target *Y*,  we name these methods as *Semi*-MIM or *Semi*-JMI respectively. Section 6 compares the performance of our suggested methods with other state-of-the-art semi-supervised feature selection methods. Before that in the next section we present applications of our work in the area of semi-supervised MB discovery.
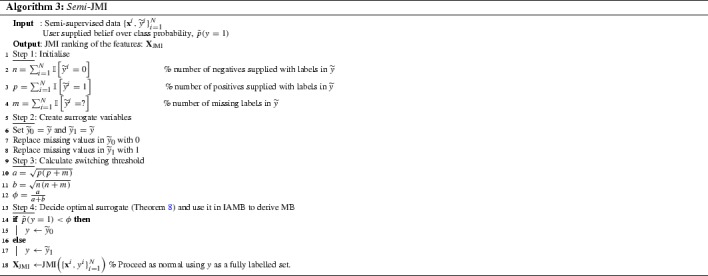


## Application 1: Semi-supervised Markov Blanket discovery

Now we will explore how to derive the MB of semi-supervised nodes. This application of our work was first presented in Sechidis and Brown (2015). Firstly, we will show how we can use surrogate variables to derive the MB of positive-unlabelled nodes, a scenario where BASSUM cannot be applied. Then we will compare the performance of our suggested method *Semi*-IAMB against a baseline method and BASSUM.

### MB discovery in positive-unlabelled learning

In this section we present how we can use our methods for testing conditional independence in PU data to derive MB despite the labelling restriction. In the PU setting, the surrogate variable ${\widetilde{Y}_0}$ is fully observed and it is identical to the labelling variable *S*. Using this surrogate instead of *Y* is a *valid* (in the sense of Definition 1) approach to test conditional independence, because of Theorem 6. This will result in the same number of *false positive errors* for the two tests, or in MB context using the surrogate variable ${\widetilde{Y}_0}$ instead of the unobservable *Y* will result in the same number of nodes falsely added to the blanket.

Now we will verify the consequences of this theorem in the context of MB discovery. We use four widely used standard benchmark networks for Markov blanket discovery taken from the Bayesian network repository.^3^ Table 2 presents the summary of these networks. For target variables we used nodes that have at least one child, one parent and one spouse in their Markov blanket. Multi-class target nodes were transformed to binary by keeping the examples with value 1 as positives and the rest of the examples formed the negative class. Furthermore, we kept the nodes that the positive class is the minority with minimum probability of 0.15. For these networks we know the true Markov blankets around each target variable and we compare them with the discovered blankets through the IAMB algorithm. For the supervised scenarios (i.e. when we used the variable *Y*) we perform 10 trials of size $N=2000$ and 5000. For each trial we sample 30 different partially labelled datasets, and the overall outcome of the partially labelled approaches was the most frequently derived Markov blanket.

**Table 2:** Networks used in Markov blanket discovery experiments

Network	Number of target nodes	Total number of nodes	Average MB length of target nodes
Alarm	5	37	5.6
Insurance	10	27	6.2
Barley	10	48	5.6
Hailfinder	20	56	4.9

**Fig. 6:**
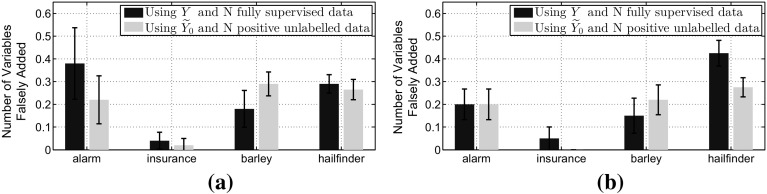
Verification of Theorem 6. This illustrates the average number of variables falsely added in MB and the 95% confidence intervals over 10 trials when we use IAMB with *Y* and ${\widetilde{Y}_0}$. **a** for total sample size $N=2000$ out of which we label only 100 positive examples and **b** for total sample size $N=5000$ out of which we label only 250 positives. **a** $N=2000,\;p({\widetilde{y}_0=1}) = 0.05$. **b** $N=5000,\;p({\widetilde{y}_0=1}) = 0.05$

As we observe from Fig. 6 using ${\widetilde{Y}_0}$ instead of *Y* in the IAMB algorithm does not result in a statistically significant difference in the false positive rate, or in MB terminology the blankets derived from these two approaches are similar in terms of the variables that were *falsely added to the blanket*.

#### Incorporating “exact” prior knowledge in sample size determination

While the surrogate approach guarantees the same number of false positive errors, a direct consequence of Theorem 7 is that using ${\widetilde{Y}_0}$ instead of *Y* results in a higher number of *false negative* errors. By using the correction factor $\kappa _{{\widetilde{Y}_0}}$ and “exact” prior knowledge over the $p(y=1)$ we can use the surrogate test for sample size determination, and decide the amount of data that we need in order to have similar performance with the unobservable fully-supervised test in terms of false negatives.

**Fig. 7:**
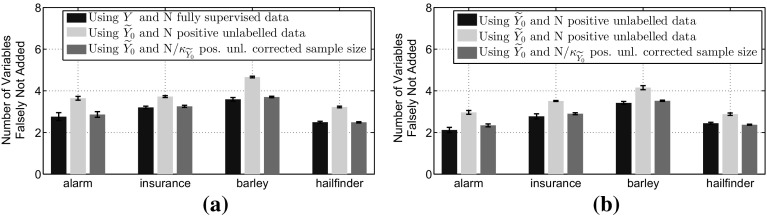
Verification of Theorem 7. This illustrates the average number of variables falsely not added to the MB and the 95% confidence intervals over 10 trials when we use IAMB with *Y* and ${\widetilde{Y}_0}$. **a** for total sample size $N=2000$ and **b** for total sample size $N=5000$. In all the scenarios we label 5% of the total examples as positives. **a** $N=2000,\;p({\widetilde{y}_0=1}) = 0.05$. **b** $N=5000,\;p({\widetilde{y}_0=1}) = 0.05$

In the MB discovery context this will result in a larger number of variables falsely not added to the predicted blanket, since we assumed that the variables were independent when in fact they were dependent. In order to verify experimentally this conclusion we will compare again the discovered blankets using ${\widetilde{Y}_0}$ instead of *Y*. As we see in Fig. 7, the number of variables that were falsely not added is higher when we are using ${\widetilde{Y}_0}$. This figure also verifies Theorem 7, where we see that the number of variables falsely removed when using the surrogate test $G(X;{\widetilde{Y}_0}|{\mathbf{Z}})$ with increased sample size $N/\kappa _{{\widetilde{Y}_0}}$ is the same as when using the unobservable test $G(X;Y|{\mathbf{Z}})$ with *N* data.

#### Evaluation of MB discovery in PU data

**Fig. 8:**
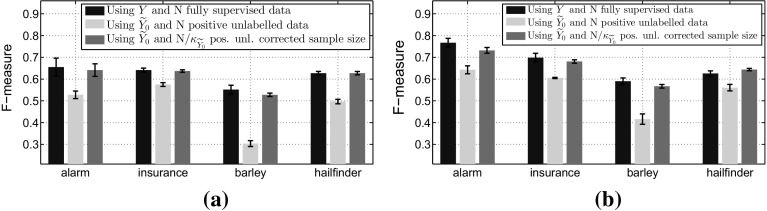
Comparing the performance in terms of *F*-measure when we use IAMB with *Y* and ${\widetilde{Y}_0}$. **a** For total sample size $N=2000$ and **b** for total sample size $N=5000$. In all the scenarios we label 5% of the total examples as positives. **a** $N=2000,\;p({\widetilde{y}_0=1}) = 0.05$. **b** $N=5000,~p({\widetilde{y}_0=1}) = 0.05$

For an overall evaluation of the derived blankets using ${\widetilde{Y}_0}$ instead of *Y* we will use the *F*-measure, which is the harmonic mean of precision and recall, against the ground truth. In Fig. 8, we observe that the assumption of all unlabelled examples to be negative gives worse results than the fully-supervised scenario, and that the difference between the two approaches gets smaller as we increase sample size. Furthermore, using the correction factor $\kappa _{{\widetilde{Y}_0}}$ to increase the sample size of the surrogate approach makes the two techniques perform similar.

### MB discovery in semi-supervised learning under class-prior-change

In this section, we will present how our approach performs in a real world problem where the class balance in the labelled set does not reflect the balance over the overall population; such situation is known as *class-prior-change* (Plessis and Sugiyama 2012), Sect. 2.3 gives more details about the assumptions behind this scenario. We compare our approach (*Semi*-IAMB) with the following two approaches: ignoring the unlabelled examples, a procedure known in statistic as *listwise deletion* (Allison 2001), or using the unlabelled data to have more reliable estimates for the marginal counts of the features, a procedure known in statistics as *available case analysis* or *pairwise deletion* (Allison 2001). The latter is followed in BASSUM (Cai et al. 2011); Sect. 2.1 gives more details about this approach and its limitations. We call the other two approaches as Listwise-IAMB and Pairwise-IAMB respectively.

**Fig. 9:**
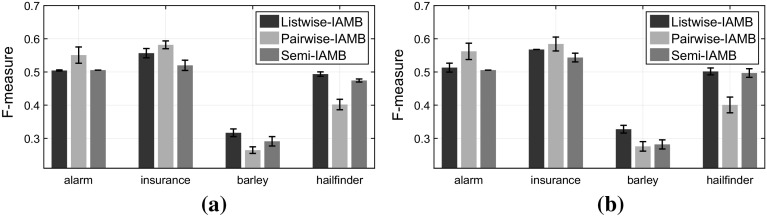
**Traditional semi-supervised (MCAR) scenario**: Comparing the performance in terms of *F*-measure when we have the same class-ratio in the labelled-set as in the overall population. **a** For sample size $N=2000$ out of which we label only 200 examples and **b** $N=5000$ out of which we label only 250 examples. **a** $N=2000,\;N_{s=1} = 200$. **b** $N=5000,\;N_{s=1} = 250$

Firstly, let us assume that the semi-supervised data are generated under the “traditional semi-supervised” scenario, where the labelled set is an unbiased sample from the overall population, or in other words the labels are MCAR. As a result, the class-ratio in the labelled set is the same to the population class-ratio: $\frac{p(y=1|s=1)}{p(y=0|s=1)} = \frac{p(y=1)}{p(y=0)}$, where the *lhs* is the class-ratio in the labelled set and in *RHS* the population class-ratio. As we observe in Fig. 9, our approach (*Semi*-IAMB) performs similarly with ignoring completely the unlabelled examples (Listwise-IAMB). As was expected, using the semi-supervised data with pairwise deletion (Pairwise-IAMB) has unpredictable performance and often performs much worse than using only the labelled examples.

**Fig. 10:**
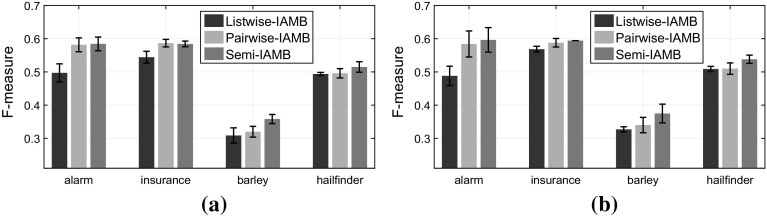
**Class-prior-change semi-supervised (MAR-C) scenario**: Comparing the performance in terms of *F*-measure when we have inverse class-ratio in the labelled-set than in the overall population. **a** For sample size $N=2000$ out of which we label only 200 examples and **b** $N=5000$ out of which we label only 250 examples. **a** $N=2000,\;N_{s=1} = 200$. **b** $N=5000,\;N_{s=1} = 250$

Now, let us assume we have semi-supervised data under the class-prior-change scenario (for more details see Sect. 2.3), or in other words the labels are MAR-C. In our simulation we sample the labelled data in order to have a class ratio in the labelled set inverse than the population ratio: $\frac{p(y=1|s=1)}{p(y=0|s=1)}=\left( \frac{p(y=1)}{p(y=0)} \right) ^{-1}$, where the *lhs* is the class-ratio in the labelled set and in *RHS* the inverse of the population class-ratio. As we observe in Fig. 10, *Semi*-IAMB performs statistically better than ignoring the unlabelled examples (Listwise-IAMB). Our approach performs better on average than the pairwise deletion, while the latter performs comparably to the listwise deletion in many settings.

Furthermore, our approach can be applied in scenarios where we have labelled examples only from one class, which cannot be handled with the other two approaches. Also, with our approach, we can control the power of our tests, which is not the case in pairwise deletion procedure. In the following section we will present the application of our work, in the area of information theoretic feature selection.

## Application 2: Semi-supervised filter feature selection

In this section we explore the performance of the *Semi*-JMI suggested in Sect. 4. Firstly, we will compare it against other information theoretic methods, and then we the state-of-the-art.

### Comparing information theoretic feature selection approaches

Firstly we will explore the performance of our suggested criteria with other information theoretic methods. We will focus on three criteria (MIM/mRMR/JMI) and their semi-supervised versions. By following Surrogate 1, or in other words using only in the labelled data ${\mathcal {D}}_L$ we rank the features using the following scores:

- MIM using $${\mathcal {D}}_L$$: $$J_{MIM}^{{\mathcal {D}}_L}(X_k)=\hat{I}(X_k;Y|s=1).$$
- JMI using $${\mathcal {D}}_L$$: $$J_{JMI}^{{\mathcal {D}}_L}(X_k)=\sum \limits _{X_j \in X_{\theta }} \!\!\! \hat{I}(X_k;Y|X_j,s=1).$$
- mRMR using $${\mathcal {D}}_L$$:$$ J_{mRMR}^{{\mathcal {D}}_L}(X_k)=\hat{I}(X_k;Y|s=1) - \frac{1}{|{\mathbf{X}}_{\theta }|}\!\!\sum \limits _{X_j \in {\mathbf{X}}_{\theta }}\!\!\!\!\hat{I}(X_k;X_j|s=1).$$

In Sect. 4.5 we suggested two semi-supervised versions of MIM and JMI:

- *Semi*-MIM: $$J_{MIM}^{{\widetilde{Y}_0}/\widetilde{Y}_1}(X_k)=\hat{I}(X_k;{\widetilde{Y}_0})~\mathrm{or}~ \hat{I}(X_k;\widetilde{Y}_1).$$
- *Semi*-JMI: $$J_{JMI}^{{\widetilde{Y}_0}/\widetilde{Y}_1}(X_k)=\sum \limits _{X_j \in X_{\theta }} \!\!\! \hat{I}(X_k;{\widetilde{Y}_0}|X_j)~\mathrm{or} \sum \limits _{X_j \in X_{\theta }} \! \! \!\!\! \hat{I}(X_k;\widetilde{Y}_1|X_j).$$

To decide between ${\widetilde{Y}_0}$ and $\widetilde{Y}_1$ we use prior knowledge and Theorem 8.

These two approaches can be also used when we have labelled information only from one class (i.e. positive-unlabelled).

In the information theoretic feature selection literature (Sect. 2.2) there is only one work for semi-supervised scenarios, MINT (He et al. 2016), which is a semi-supervised version of the mRMR criterion. Thus, we will explore how our suggested approaches behave in comparison with the following mRMR based method:

- MINT: $$ J_{mRMR}^{MINT}(X_k)=\hat{I}(X_k;Y|s=1) - \frac{1}{|{\mathbf{X}}_{\theta }|}\sum \limits _{X_j \in {\mathbf{X}}_{\theta }}\hat{I}(X_k;X_j).$$

#### Exploring the consistency of the selected subsets

An interesting question to explore is “how do the features selected through the semi-supervised approaches differ from the ones that we would have by using the unobservable target variable *Y*?” To evaluate the performance of the different approaches, we will measure the similarity between the top-ten features that are returned by the semi-supervised approaches and the features that we would have if we had a full supervision over the target. If the consistency is high it indicates that the selected set is similar. We will measure this similarity by Kuncheva’s *consistency index* (Kuncheva 2007), which recently has been shown to have several nice properties (Nogueira and Brown 2016).

Table 3 gives details over the seven datasets that we use in our experiments. Multi-class datasets transformed to binary by 1-vs-all. The features within each data set have a variety of types some categorical, and some numerical. In the information theoretic feature selection step, numeric features were discretized into five bins using an equal-width strategy. These are fully-supervised datasets and we sample them to generate semi-supervised versions by labeling 25% of the examples.

**Table 3:** Datasets used in the feature selection experiments

Dataset	# Examples	# Features	$\hat{p}(y=1)$
krvskp	3196	36	0.52
landsat	6435	36	0.24
musk2	6598	166	0.15
semeion	1593	256	0.50
spambase	4601	57	0.39
splice	3175	60	0.24
waveform	5000	40	0.34

**Fig. 11:**
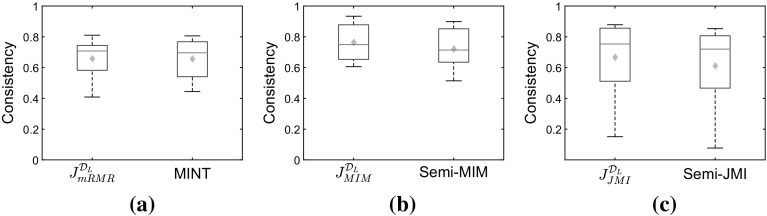
**MCAR**: Kuncheva’s Consistency index between the feature subsets returned through fully-supervised mRMR/MIM/JMI and the ones returned by using the partially labelled approaches. In this graph we present box plots and expected values (*diamonds*) across the seven datasets, while in each dataset we average the index over 30 semi-supervised versions. **a** mRMR. **b** MIM. **c** JMI

Firstly, we sample the labelled set under the traditional semi-supervised scenario MCAR. Figure 11 shows that the approaches that use both labelled and unlabelled data—MINT, *Semi*-MIM, *Semi*-JMI—perform very similar with the approaches that use only the labelled data –$J_{mRMR}^{\mathcal {D}_L}/J_{MIM}^{\mathcal {D}_L}/J_{JMI}^{\mathcal {D}_L}$.

**Fig. 12:**
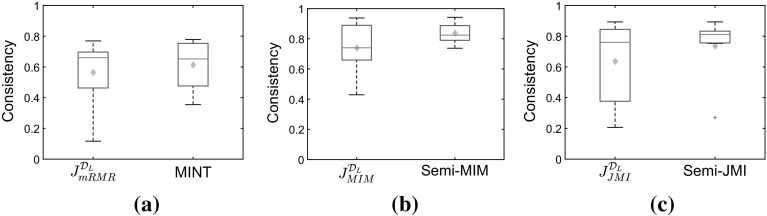
**MAR-C**: Kuncheva’s Consistency index between the feature subsets returned through fully-supervised mRMR/MIM/JMI and the ones returned by using the partially labelled approaches. In this graph we present box plots and expected values (*diamonds*) across the seven datasets, while in each dataset we average the index over 30 semi-supervised versions. **a** mRMR. **b** MIM. **c** JMI

Then, we generate semi-supervised datasets with class-prior-change where the labels are MAR-C. We label the examples in such a way that in the labelled set we have two times more positive than negative examples. Figure 12 shows that the approaches that use both labelled and unlabelled data—MINT, *Semi*-MIM, *Semi*-JMI—outperform the approaches that use only the labelled set –$J_{mRMR}^{\mathcal {D}_L}/J_{MIM}^{\mathcal {D}_L}/J_{JMI}^{\mathcal {D}_L}$. This trend is more obvious in our suggested semi-supervised criteria, *Semi*-MIM and *Semi*-JMI, than in MINT. This result verifies the fact that our suggested method are suitable for both MCAR and MAR-C semi-supervised scenarios, while MINT is only for MCAR.

#### Exploring the misclassification error

In this section we will explore the performance of the semi-supervised criteria in terms of their misclassification error. We used 10 train/test splits with 50% of the data used for training and 50% for testing. To generate the semi-supervised data we labelled 25% of the training examples—$p(s=1) = 0.25$. We select the five most important features using different semi-supervised criteria. Then we use the selected features and the training data to build a *k*-nearest neighbor classifier ($k=3$), since this classifier does not make any probabilistic assumptions (Brown et al. 2012), and we measure the accuracy of the classifiers in the testing data.

**Fig. 13:**
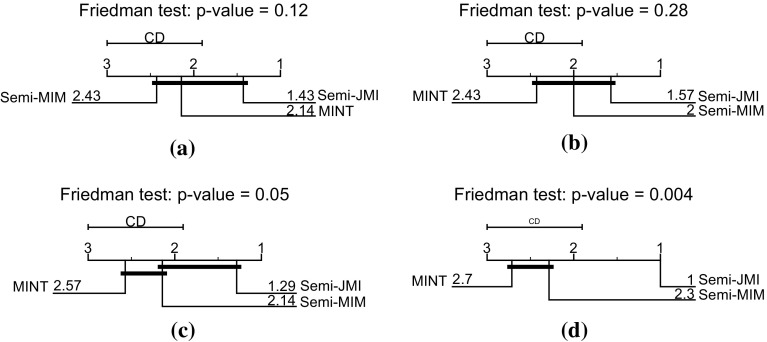
**Critical diagrams**: Comparison of **information theoretic** semi-supervised feature selection methods for different semi-supervised scenarios. We generate the semi-supervised datasets under the class-prior-change scenario by randomly under or over-sampling the positive class, such that the probability of a labelled example being positive—$p(y=1|s=1)$—to be $(1- c) \times p(y=1)$ or $(1+ c) \times p(y=1)$ respectively. **a** MCAR (which means $c=0$), **b** MAR-C with $c=0.25$, **c** MAR-C with $c=0.50$ and **d** the most extreme MAR-C with $c=0.75$. For the Nemenyi post-hoc test we set the significance level to be 0.10. **a** MCAR $\equiv$ (MAR-C with $c=0$). **b** MAR-C with $c=0.25$. **c** MAR-C with $c=0.50$. **d** MAR-C with $c=0.75$

Firstly, we will examine under the traditional semi-supervised scenario where the labelled set is an unbiased sample from the overall population. Table 4 presents the misclassification error over the 10 train/test splits. As we observe, there was no clear winner, but on average our suggested semi-supervised criterion *Semi*-JMI achieves better performance. To explore the statistical significance of our results we analysed the ranks of the three methods by using a Friedman test with the Nemenyi post-hoc test. Figure 13a presents the *critical difference diagrams*, introduced by Demšar (2006), where groups of methods that are not significantly different (at $\alpha = 0.10$) are connected. As this figure shows, *Semi*-JMI performs better on average but with no statistical significance.

**Table 4:** Comparisons of the misclassification error using features derived from different **information theoretic** semi-supervised criteria when the labels are **MCAR**

Dataset	MINT (He et al. 2016)	*Semi*-MIM (our approach)	*Semi*-JMI (our approach)
krvskp	0.078 ± 0.019	0.080 ± 0.021	**0.072** ± 0.020
landsat	0.042 ± 0.004	0.101 ± 0.072	**0.023** ± 0.003
musk2	0.097 ± 0.008	0.080 ± 0.005	**0.078** ± 0.012
semeion	0.149 ± 0.018	0.185 ± 0.108	**0.145** ± 0.017
spambase	**0.179** ± 0.018	0.198 ± 0.023	0.200 ± 0.022
splice	**0.044** ± 0.004	**0.044** ± 0.004	**0.044** ± 0.004
waveform	0.214 ± 0.009	0.191 ± 0.007	**0.182** ± 0.006

For each dataset we present the average error and the standard deviation over the 10 trials, while bold indicates the lowest average error

Then, we generate semi-supervised datasets under the class-prior-change scenario by randomly under or over-sampling the positive class, such that the probability of a labelled example being positive—$p(y=1|s=1)$—to be $0.5\times p(y=1)$ or $1.5 \times p(y=1)$ respectively. Table 5 presents the average misclassification error and the 95% confidence intervals. As we observe our suggested approach, *Semi*-JMI, which takes into account relevancy, redundancy and redundancy, outperforms all the other approaches. Furthermore, Fig. 13c shows that the difference between *Semi*-JMI and MINT is statistically significant.

**Table 5:** Comparisons of the misclassification error using features derived from different **information theoretic** semi-supervised criteria when the labels are **MAR-C**

Dataset	MINT (He et al. 2016)	*Semi*-MIM (our approach)	*Semi*-JMI (our approach)
krvskp	0.108 ± 0.067	0.082 ± 0.020	**0.079** ± 0.021
landsat	0.044 ± 0.004	0.102 ± 0.078	**0.023** ± 0.004
musk2	0.099 ± 0.008	0.085 ± 0.006	**0.082** ± 0.006
semeion	0.168 ± 0.040	0.147 ± 0.013	**0.142** ± 0.012
spambase	**0.171** ± 0.018	0.185 ± 0.020	0.185 ± 0.019
splice	0.065 ± 0.012	0.049 ± 0.011	**0.044** ± 0.004
waveform	0.217 ± 0.005	0.191 ± 0.009	**0.178** ± 0.008

For each dataset we present the average error and the standard deviation over the 10 trials, while bold indicates the lowest average error

Figure 13 shows the performance of the three semi-supervised feature selection methods for different labelling scenarios from MCAR in Fig. 13a to extreme MAR-C in Fig. 13d. Our semi-supervised JMI version always outperforms on average the rest of the methods, and this trend is more obvious and statistically significant when we have strong class-dependent labelling, or in other words the class ratio in the labelled set is much different than the population class ratio.

### Comparison with state-of-the-art semi-supervised feature selection methods

In this section we compare our best performing proposed method (*Semi*-JMI) with state-of-the-art methods in the semi-supervised feature selection (Ang et al. 2016). The comparison will be in terms of misclassification error (using 3-nearest neighbour classifier) and the experimental setting is the same as in Sect. 6.1.2.

Zhao and Liu (2007) proposed sSelect, one of the earliest algorithms for semi-supervised feature selection, based on spectral graph theory. There is a great amount of literature dedicated to methods motivated by different perspectives; for instance Sheikhpour et al. (2017) have recently published a thorough survey that summarises all of these methods. For our experiments we considered methods as technically diverse as possible, here we provide a short description:

- FW-SemiFS (Ren et al. 2008): A wrapper forward semi-supervised feature selection, which uses the unlabeled examples to extend the initial labeled training set in a process is similar to “co-training”. For the co-training we used a 3-nearest neighbour classifier.
- CLS (Benabdeslem and Hindawi 2011): A method based on a semi-supervised version of the Laplacian score. This approach has a graph based formulation, which has been the basis of other feature selection methods, such as sSelect. One key assumption behind this approach is that both labelled and unlabelled examples are sampled from the same distribution, which only holds in MCAR.
- SemiFS (Liu et al. 2013): A noise insensitive trace ratio criterion for selecting relevant features using both labeled and unlabeled data. CLS and SemiFS are more suitable for numerical features.
- MINT (He et al. 2016): A semi-supervised version of mRMR, details in Sect. 6.1.
- RRPC (Xu et al. 2016): A max-relevance and min-redundancy criterion based on Pearson’s correlation (RRPC) coefficient. One advantage of this coefficient is that it can be used to measure the correlation between numerical features, but the main disadvantage is that it detects only linear correlations.

**Fig. 14:**
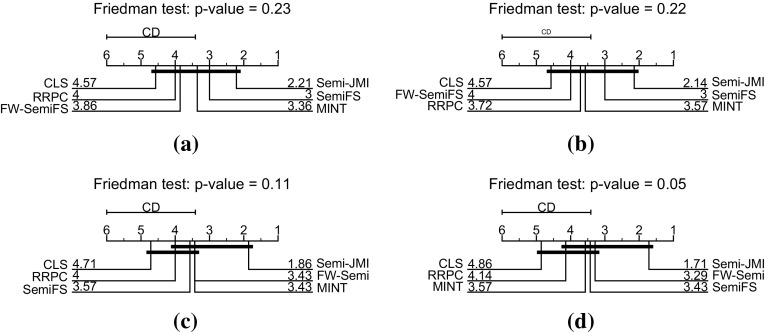
**Critical diagrams**: Comparison of semi-supervised feature selection methods for different semi-supervised scenarios. Experimental setting same as for Fig. 13. **a** MCAR $\equiv$ (MAR-C with $c=0$). **b** MAR-C with $c=0.25$. **c** MAR-C with $c=0.50$. **d** MAR-C with $c=0.75$

Firstly, we compare the performance of the algorithms when the labelled set is an unbiased sample (MCAR). Table 6 shows that there is no clear winner, but on average our suggested semi-supervised criterion *Semi*-JMI seems to have better performance on average. This can be seen in the critical differences diagram in Fig. 14a.

**Table 6:** Comparisons of the average misclassification error using features derived from different semi-supervised feature selection methods when the labels are **MCAR**

Dataset	FW-SemiFS	CLS	SemiFS	RRPC	MINT	*Semi*-JMI
krvskp	0.366	0.473	0.118	0.358	0.078	**0.072**
landsat	0.182	0.248	**0.023**	0.074	0.042	**0.023**
musk2	0.064	**0.061**	0.093	0.079	0.097	0.078
semeion	0.201	**0.141**	**0.141**	0.188	0.149	0.145
spambase	**0.143**	0.299	0.212	0.145	0.179	0.200
splice	0.167	0.258	0.065	0.117	**0.044**	**0.044**
waveform	0.194	0.424	0.194	0.237	0.214	**0.182**

Experimental setting same as for Table 4

Then, we generate the semi-supervised data with biased labelled set under the class-prior-change scenario (MAR-C). Table 7 presents the average misclassification error. As we observe our suggested semi-supervised *Semi*-JMI outperforms the other approaches in most of the datasets. Figure 14c verifies this since our method is ranked first.

**Table 7:** Comparisons of the misclassification error using features derived from different semi-supervised feature selection methods when the labels are **MAR-C**

Dataset	FW-SemiFS	CLS	SemiFS	RRPC	MINT	*Semi*-JMI
krvskp	0.373	0.471	0.119	0.368	0.108	**0.079**
landsat	0.129	0.224	0.024	0.068	0.044	**0.023**
musk2	0.067	**0.060**	0.093	0.080	0.099	0.082
semeion	0.146	0.147	0.184	0.197	0.168	**0.142**
spambase	**0.141**	0.278	0.218	0.150	0.171	0.185
splice	0.278	0.268	0.097	0.122	0.065	**0.044**
waveform	0.197	0.423	0.192	0.245	0.217	**0.178**

Experimental setting same as for Table 5

Finally, Fig. 14 shows the performance of the methods for different labelling scenarios from MCAR in Fig. 14a to extreme MAR-C in Fig. 14d. Our proposed method, *Semi*-JMI, always ranked first, and this trend is more obvious when we have strong class-dependent labelling, or in other words the probability of the class in the labelled $p(y=1|s=1)$ set is very different from the actual class probability $p(y=1)$.

## Conclusions and future work

We presented a study of two extremely simple *inference-free* approaches to information theoretic feature selection in semi-supervised scenarios.

### Summary of contributions

In the beginning of this work, we posed two tangled questions on testing and ranking of features. To give sensible answers in semi-supervised scenarios, we modelled the underlying mechanism of missing labels with the two main assumptions used in the literature: MCAR and MAR-C. To answer our questions in an entirely classifier-independent manner, we derived from the observed data two *surrogate* approaches and we analysed what the consequences would be of using these surrogates instead of the unobservable target labels in the different partially labelled scenarios. We had the following contributions:

1. We derived ways for performing valid and informed hypothesis testing in semi-supervised environments (Sect. 3). The outcome of our analysis is a methodology that enables to incorporate some “soft” knowledge in order to decide which surrogate approach is optimal.
2. Building upon our theoretical results for semi-supervised hypothesis testing we proposed a novel Markov blanket discovery algorithm *Semi*-IAMB. Section 5 showed how to use this algorithm for discovering the MB around positive-unlabelled and semi-supervised targets.
3. We derived ways to use surrogate variables in order to rank the features as if we had fully supervised data (Sect. 4).
4. Using our theoretical findings, we proposed an algorithm for semi-supervised feature ranking, *Semi*-JMI, with several nice properties (i.e. captures relevancy, redundancy and conditional redundancy). Section 6 showed experimentally that our algorithm outperforms previously suggested approaches, especially when the labels are missing-not-at-random.

### Future work

There are two interesting research directions:

- **Extending to numerical features**
  All of the theoretical results about ranking, Sect. 4, hold also for numerical features. From our findings in Sect. 3 the results about the *validity* (Theorems 1, 3 and 6) hold also for numerical features. One possible way to extend our results about the *informedness* (Theorems 2, 4 and 7) to numerical features is the following. In our work we exploited the relationship between *G*-test and mutual information, when the features are numerical, and under some assumptions, there is a relationship between the unpaired *t*-test and the point-biserial correlation coefficient (Rosenthal et al. 2000). Exploiting this connection, it may be useful step in order to completely extend our methodology to numerical features.
- **Extending to other types of missingness**
  A future research direction could be to explore how we can use our methodology to other types of missingness. For example another assumption used in the semi-supervised learning is when the missingness mechanism depends directly only on the features or in other words the labelling of an example is conditionally independent of the class, given the feature values. This scenario is known in missing data literature as missing at random (MAR) (Moreno-Torres et al. 2012). The importance of this assumption is also presented in the framework of semi-supervised regression by Lafferty and Wasserman (2007). Another possible direction is to explore under which assumptions over the model and what type of prior knowledge do we need in order to perform feature selection when the labels are MNAR. In this scenario the missingness mechanism depends directly on both the features and the target variable—one possible strategy can be to decompose the problem into MAR-C and MAR sub-problems. Furthermore, we can explore ways to deal with missing data both in features and in labels. One way is to combine our work with a recently suggested framework for feature selection when we have missing or misclassified features (Sechidis et al. 2017).

## Tutorial on information theoretic testing and estimation

In filter feature selection, the features ${\mathbf{X}}$ are ranked by choosing those that share the most information with the class label *Y*. In machine learning the two main ways to measure this information is by using Shannon’s Mutual Information (MI) (Cover and Thomas 2006) or Squared-loss Mutual Information (SMI) (Sugiyama 2012). The MI is the *Kullback-Leibler divergence* between the joint *p*(*x*, *y*) and the product of the marginals *p*(*x*)*p*(*y*), $I(X;Y) = D_{KL}(p(x,y)||p(x)p(y)),$ while the SMI is the *Pearson divergence* between the joint and the product of the marginals. These two quantities can be estimated from sample data using the following *maximum-likelihood* estimators:

5

6

where $\hat{p}(x,y)$ is the maximum-likelihood estimate of the probability that the random variable *X* takes on the value *x* from its alphabet $\mathcal {X}$ and *Y* takes on $y\in \mathcal {Y}$, while $\hat{p}(x)$ and $\hat{p}(y)$ the estimates for the marginal probabilities. It can be proved that the SMI ${I}_{2}(X;Y)$ is a second order Taylor series approximation of Shannon’s MI *I*(*X*; *Y*).

The estimates of the mutual information can be seen as measures of *effect* size when we want to quantify the dependency between random variables, and have several nice properties. Firstly, they are non-negative quantities which take their minimum zero value when the random variables are independent. Furthermore, MI can be associated with both upper and lower bounds on the Bayes error. Brown et al. (2012) present an extensive discussion of this in the context of feature selection, including various heuristics which provide approximations for high dimensional data, resulting in a unifying theoretical framework derived from a simple probabilistic model. A crucial term in this framework is the conditional MI:

This can be thought of as the information shared between *X* and *Y* after the values of a subset of features ${\mathbf{Z}}\subseteq {\mathbf{X}} \backslash X$, are revealed. As we will show in Sect. 2.2, using the unconditional mutual information captures only the relevancy with the target, while using conditional mutual information we can capture also the redundancy between the features (Brown et al. 2012).

Answering whether two random variables are independent or not requires us to threshold the value of the estimated mutual information. To derive such a threshold we will use the asymptotic distribution of the estimator and a hypothesis testing procedure. By following this procedure we will have an informed decision and a control over the two possible errors: concluding independence where in fact there is a dependence (a false negative, or type-II error), or the opposite, concluding dependence where in fact there is none (a false positive, or type-I error). In our work, we will focus on the *Neyman-Pearson* procedure for hypothesis testing (Berger 2003) and we will explore two widely used tests of independence in categorical data: the *G*-test and the -test (Cressie and Read 1989). Both of them have been widely used in machine learning, for example in structure learning of Bayesian networks (Spirtes et al. 2001).

The *G*-test is a generalised likelihood ratio test, where the test statistic can be calculated from data counts arranged in a contingency table. We denote by $o_{x,y,\mathbf{z}}$ the observed count of the number of times the random variable *X* takes on the value $x \in \mathcal {X}$, *Y* takes on $y\in \mathcal {Y}$ and $\mathbf{Z}$ takes on , where ${\mathbf{z}}$ is a vector of values when we condition on more than one variable. Furthermore, $o_{x,.,{\mathbf{z}}}$, $o_{.,y,{\mathbf{z}}}$ and $o_{.,.,{\mathbf{z}}}$ denote the observed marginal counts. The estimated expected frequency of $(x,y,{\mathbf{z}})$, assuming *X*, *Y* are conditionally independent given ${\mathbf{Z}}$, is given by $e_{x,y,{\mathbf{z}}} = \frac{o_{x,.,{\mathbf{z}}}o_{.,y,{\mathbf{z}}}}{o_{.,.,{\mathbf{z}}}}=\hat{p}(x|{\mathbf{z}})\hat{p}(y|{\mathbf{z}})o_{.,.,{\mathbf{z}}}$. To calculate the *G*-statistic we use the formula:

7

From this expression we see the relationship between the *G*-statistic and MI, and the latter can be seen as the natural unit of *effect size* for the *G*-test (Rosenthal et al. 2000). Under the null hypothesis ($H_0$) that *X* and *Y* are statistically independent given ${\mathbf{Z}}$, the *G*-statistic is known to be asymptotically $\chi ^2$-distributed, with  degrees of freedom (Agresti 2013). For a given dataset, we calculate (7) and then the *p*-value, which is $1 - F({G(X;Y|{\mathbf{Z}})})$, where *F* is the CDF of the $\chi ^2$-distribution. The *p*-value represents the probability of obtaining a test statistic equal or more extreme than the observed one, given that the null hypothesis holds. After calculating this value, we check to see whether it exceeds a user specified significance level $\alpha$. If ${p}{\text{- }}\mathrm{value} \le \alpha$, we reject the null hypothesis of independence.

While the user specified significance level defines the probability of *type I error* ($\alpha$), in order to explore the probability of *type II error* ($\beta$), we should perform a *power analysis* (Cohen 1988). The *power* of a test is the probability that the test will reject the null hypothesis when the alternative hypothesis is true—or in practical machine learning terms, the probability of correctly selecting a relevant feature. This is also known as the *true positive rate*, or the probability of *not* committing a type II error. One important usage of a-priori power analysis is *sample size determination*. In this prospective procedure we specify the probability of type I error (e.g. $\alpha =0.05$), the desired probability of type II error (e.g. $\beta = 0.01$ or $power = 0.99$) and the desired effect size that we want to observe (i.e. expressed in terms of *I*(*X*; *Y*) for the *G*-test), and we can determine the minimum number of examples (*N*) that we need to detect that effect. However, to do this we need a test statistic with a known distribution under the alternative hypothesis. It is known that the *G*-statistic has a large-sample *non-central* $\chi ^2$ distribution under the alternative hypothesis (Agresti 2013, Section 6.6.4), with the same degrees of freedom as in the null distribution. The non-centrality parameter –$\lambda _{G(X;Y|{\mathbf{Z}})}$—has the same form as the *G*-statistic, but with sample values replaced by population values, $\lambda _{G(X;Y|{\mathbf{Z}})} = 2 N I(X;Y|{\mathbf{Z}})$.

The other popular way to test independence between categorical random variables is by using the -test (Cressie and Read 1989), where we calculate the -statistic as . This test is closely associated with the *G*-test, since the -statistic is the second order approximation of the *G*-statistic, making the *G* and -test asymptotically equivalent (Agresti 2013). The closer we are to independence, or—equivalently—when the effects are small, the better the approximation will be. These are the effects of main interest, since when we have larger effects differentiating between relevant/irrelevant features is more trivial. In our analysis, we will make use of the asymptotic equivalence between the two versions for testing independence and the for estimating mutual information. Both of these two versions will turn out to be particularly important in the context of *hypothesis testing* and *feature ranking* in partially labelled data. More details about the relationship between hypothesis testing and feature selection can be found in Sechidis (2015).

## Proofs and sketches of proofs

### Theorem 1

When the labels are MCAR, the following equations hold, which are useful for our proofs.

8 $$\begin{aligned} p(x,y|s=1)= & {} p(x,y) , \end{aligned}$$

9 $$\begin{aligned} p(x|s=1)= & {} p(x) ,\end{aligned}$$

10 $$\begin{aligned} p(y|s=1)= & {} p(y) . \end{aligned}$$

$\mathbf{Surrogate~1}~(\mathcal {D}_L)$: To prove , we need to prove:

$$\begin{aligned} p(x,y|s=1) = p(x|s=1)p(y|s=1) \Leftrightarrow p(x,y) = p(x)p(y) ~~~~\forall ~~x~\in ~\mathcal {X}~~and ~~y~\in ~\mathcal {Y}. \end{aligned}$$

The proof is straightforward:

$$\begin{aligned} p(x,y|s=1) = p(x|s=1)p(y|s=1) \mathop \Leftrightarrow \limits ^{(8), (9), (10)} p(x,y) = p(x)p(y). \end{aligned}$$

$\mathbf{Surrogate~2}~({\widetilde{Y}_0})$ and $\mathbf{Surrogate~3}~(\widetilde{Y}_1)$: We can prove that these surrogate approaches are valid by following the same methodology, or we can read that the independence relationships hold from the *m*-graph of MCAR in Fig. 2a. $\square$

### Theorem 2

$\mathbf{Surrogate~1}~(\mathcal {D}_L)$: The non-centrality parameter of this surrogate test is equal to

$\lambda _{G(X;Y|s=1)} = 2 N_{s=1} I(X;Y|s=1)$, where $N_{s=1}$ represents the size of the labelled set. With straightforward calculus, and using Eqs. (8)–(10), we can show that when the labels are MCAR it holds $I(X;Y|s=1) = I(X;Y)$. We can write the non-centrality parameter of the surrogate approach as:

$$\begin{aligned} \lambda _{G(X;Y|s=1)} = 2 N_{s=1} I(X;Y) \Leftrightarrow \lambda _{G(X;Y|s=1)} = \frac{N_{s=1}}{N} 2 N I(X;Y) \end{aligned}$$

The fraction $\frac{N_{s=1}}{N}$ represents the probability of labelling an example $p(s=1)$, while 2*NI*(*X*; *Y*) is the non-centrality parameter of the unobservable test. Thus $\lambda _{G(X;Y|s=1)} = p(s=1) \lambda _{G(X;Y)}$, and the correction factor is $\kappa = p(s=1)$.

$\mathbf{Surrogate~2}~({\widetilde{Y}_0})$: In order to prove that relationship we will use the result of Shelby (1974) that when we assume local alternatives the $X^2$ and the *G*-test have the same asymptotic power (Shelby 1974, p. 109), in other words their non-centrality parameters converge to a common value as $N \rightarrow \infty$ (Agresti 2013, Section 16.3.5). So instead of exploring the relationship of the non-centrality parameters for the *G*-tests between $X,{\widetilde{Y}_0}$ and *X*, *Y*, we can explore the relationship between the non-centrality parameters of the $X^2$-tests between $X,{\widetilde{Y}_0}$ and *X*, *Y*. The non-centrality parameter of this $X^2$ surrogate test is equal to $\lambda _{X^2(X;{\widetilde{Y}_0})} = 2 N I_2(X;{\widetilde{Y}_0})$. With straightforward calculus and using Eqs. (8)–(10), we can show that when the labels are MCAR it holds $I_2(X;{\widetilde{Y}_0}) = \frac{1-p(y=1)}{1-p(y=1)p(s=1)}p(s=1)I_2(X;Y)$. We can write the non-centrality parameter of the surrogate approach as:

$$\begin{aligned} \lambda _{X^2(X;{\widetilde{Y}_0})}= & {} \frac{1-p(y=1)}{1-p(y=1)p(s=1)}p(s=1) 2 N I_2(X;Y) \Leftrightarrow \\ \lambda _{X^2(X;{\widetilde{Y}_0})}= & {} \frac{1-p(y=1)}{1-p(y=1)p(s=1)}p(s=1) \lambda _{X^2(X;Y)} \end{aligned}$$

By using the result that the non-centrality parameters for the $X^2$ and *G*-test converge to a common value, we can re-write the above relationship using the non-centrality parameter of the *G*-test

$$\begin{aligned} \lambda _{G(X;{\widetilde{Y}_0})}= & {} \frac{1-p(y=1)}{1-p(y=1)p(s=1)}p(s=1) \lambda _{G(X;Y)} \end{aligned}$$

Thus the correction factor is $\kappa = \frac{1-p(y=1)}{1-p(y=1)p(s=1)}p(s=1)$.

$\mathbf{Surrogate~3}~(\widetilde{Y}_1)$: We can prove this correction factor by following the same methodology as for ${\widetilde{Y}_0}$. This time it holds $I_2(X;\widetilde{Y}_1) = \frac{1-p(y=0)}{1-p(y=0)p(s=1)}p(s=1)I_2(X;Y)$, and as a result the correction factor is $\kappa = \frac{1-p(y=0)}{1-p(y=0)p(s=1)}p(s=1)$. $\square$

### Theorem 3

When the labels are MAR-C, the following equations hold, which are useful for our proofs.

11 $$\begin{aligned} p(x|y,s=1)= & {} p(x|y) , \end{aligned}$$

12 $$\begin{aligned} p(x|{\widetilde{y}_0=1})= & {} p(x|y=1), \end{aligned}$$

13 $$\begin{aligned} p(x|\widetilde{y}_1=0)= & {} p(x|y=0). \end{aligned}$$

$\mathbf{Surrogate~1}~(\mathcal {D}_L)$: To prove , we need to prove:

$$\begin{aligned} p(x,y|s=1) = p(x|s=1)p(y|s=1) \Leftrightarrow p(x,y) = p(x)p(y) ~~~~\forall ~~x~\in ~\mathcal {X}~~and ~~y~\in ~\mathcal {Y}. \end{aligned}$$

The proof is straightforward by using Eq. (11) and Dawid’s (1979) definition of independence, Eq. (IIb) in Dawid (1979). A similar proof is given for the conditional independence by Didelez et al. (2010, Theorem 6).

$\mathbf{Surrogate~2}~({\widetilde{Y}_0})$ and $\mathbf{Surrogate~3}~(\widetilde{Y}_1)$: We can prove that these surrogates are valid by following the same methodology, or we can read that the independence relationships hold from the *m*-graph of MARC in Fig. 2b. For completeness we will give the analytical proof for one scenario, i.e. the Surrogate 2. To prove , we need to prove that

Since the random variable *Y* is binary it is sufficient to prove this for the two classes. So for the first class we have

$$\begin{aligned} p(x,{\widetilde{y}_0=1}) = p(x)p({\widetilde{y}_0=1})\Leftrightarrow & {} p(x|{\widetilde{y}_0=1}) = p(x) \mathop \Leftrightarrow \limits ^{(12)} \\ p(x|y=1) = p(x)\Leftrightarrow & {} p(x,y=1) = p(x) p(y=1). \end{aligned}$$

Using the above result for the first class, we will prove it also for the second class

$$\begin{aligned}&p(x,{\widetilde{y}_0=0}) = p(x)p({\widetilde{y}_0=0}) \Leftrightarrow p(x) - p(x,{\widetilde{y}_0=1}) = p(x)(1-p({\widetilde{y}_0=1})) \Leftrightarrow \\&p(x,{\widetilde{y}_0=1}) = p(x)p({\widetilde{y}_0=1}) \Leftrightarrow p(x,y=1) = p(x)p(y=1) \Leftrightarrow \\&p(x) - p(x,y=0) = p(x) (1-p(y=0)) \Leftrightarrow p(x,y=0) = p(x)p(y=0). \end{aligned}$$

$$\square $$

### Theorem 4

$\mathbf{Surrogate~1}~(\mathcal {D}_L)$: In order to prove that this test is informed we need to re-express the non-centrality parameter of the unobservable test $\lambda _{G(X;Y)}$ as $\kappa \lambda _{G(X;Y|s=1)}$. To do so, we need to re-express *I*(*X*; *Y*) as $\kappa I(X;Y|s=1)$, or to re-express $I_2(X;Y)$ as $\kappa I_2(X;Y|s=1)$. But this is not possible when the labels are MAR-C. $\mathbf{Surrogate~2}~({\widetilde{Y}_0})$: In order to prove this relationship, we will use again the result that when we assume local alternatives the $X^2$ and the *G*-test have the same asymptotic power. So instead of exploring the relationship of the non-centrality parameters for the *G*-tests between $X,{\widetilde{Y}_0}$ and *X*, *Y*, we can explore the relationship between the non-centrality parameters of the $X^2$-tests between $X,{\widetilde{Y}_0}$ and *X*, *Y*. The non-centrality parameter of this $X^2$ surrogate test is equal to $\lambda _{X^2(X;{\widetilde{Y}_0})} = 2 N I_2(X;{\widetilde{Y}_0})$. With straightforward calculus, and using Eq. (12), we can show that when the labels are MAR-C it holds $I_2(X;{\widetilde{Y}_0}) = \frac{1-p(y=1)}{p(y=1)} \frac{p({\widetilde{y}_0=1})}{1-p({\widetilde{y}_0=1})}I_2(X;Y)$. We can write the non-centrality parameter of the surrogate approach as:

$$\begin{aligned} \lambda _{X^2(X;{\widetilde{Y}_0})}= & {} \frac{1-p(y=1)}{p(y=1)} \frac{p({\widetilde{y}_0=1})}{1-p({\widetilde{y}_0=1})} 2 N I_2(X;Y) \Leftrightarrow \\ \lambda _{X^2(X;{\widetilde{Y}_0})}= & {} \frac{1-p(y=1)}{p(y=1)} \frac{p({\widetilde{y}_0=1})}{1-p({\widetilde{y}_0=1})} \lambda _{X^2(X;Y)}. \end{aligned}$$

By using the result that the non-centrality parameters for the $X^2$ and *G*-test converge to a common value, we can re-write the above relationship using the non-centrality parameter of the *G*-test

$$\begin{aligned} \lambda _{G(X;{\widetilde{Y}_0})}= & {} \frac{1-p(y=1)}{p(y=1)} \frac{p({\widetilde{y}_0=1})}{1-p({\widetilde{y}_0=1})}p(s=1) \lambda _{G(X;Y)}. \end{aligned}$$

Thus the correction factor is $\kappa _{{\widetilde{Y}_0}} = \frac{1-p(y=1)}{p(y=1)} \frac{p({\widetilde{y}_0=1})}{1-p({\widetilde{y}_0=1})}$.

$\mathbf{Surrogate~3}~(\widetilde{Y}_1)$: We can prove this correction factor by following the same methodology as for ${\widetilde{Y}_0}$. This time, by Eq. (13), we can prove that $I_2(X;\widetilde{Y}_1) = \frac{1-p(y=0)}{p(y=0)} \frac{p(\widetilde{y}_1=0)}{1-p(\widetilde{y}_1=0)}I_2(X;Y)$, and as a result the correction factor is $\kappa _{\widetilde{Y}_1} = \frac{1-p(y=0)}{p(y=0)} \frac{p(\widetilde{y}_1=0)}{1-p(\widetilde{y}_1=0)}$. $\square$

### Theorem 6

$\mathbf{Surrogate~1}~(\mathcal {D}_L)$: A proof can be found in Didelez et al. (2010, Theorem 6).

$\mathbf{Surrogate~2}~({\widetilde{Y}_0})$: To prove this theorem, we will use the following useful lemma.

#### Lemma 1

When the labels are MAR-C, the following equations hold, for any subset of features

#### Proof

To prove this Lemma we will start from the *RHS* of the desired equation:

Then by using the Bayes theorem and the chain rule we get:

Because of MAR-C assumption:

14 $$\begin{aligned} p({\widetilde{y}_0=1}|y=1,x,{\mathbf{z}})&= p({\widetilde{y}_0=1}|y=1,{\mathbf{z}}) \end{aligned}$$

As a result the last expression becomes:

This finishes the proof of this lemma, since we derived the *lhs* of the desired equation. An interesting point to clarify is that Eq. (14) holds for any subset of features. To show that, without loss of generality, let us assume that the entire set of features ${\mathbf{x}}$ consists of the variables $x,{\mathbf{z}}$ and ${\mathbf{w}}$, where *x* is a single variable and ${\mathbf{z}}$, ${\mathbf{w}}$ sets of variables. The $x,{\mathbf{z}}$ and ${\mathbf{w}}$ can be created by any feature combination as long their intersection is the empty set and their union is the entire feature space. Now we can re-write the MAR-C assumption as:

$$\begin{aligned} p({\widetilde{y}_0=1}|y=1,{\mathbf{x}})&= p({\widetilde{y}_0=1}|y=1) \Leftrightarrow \\ p({\widetilde{y}_0=1}|y=1, x,{\mathbf{z}},{\mathbf{w}})&= p({\widetilde{y}_0=1}|y=1) \Leftrightarrow \\ p({\widetilde{y}_0=1},x,{\mathbf{z}},{\mathbf{w}}|y=1)&= p({\widetilde{y}_0=1}|y=1)p(x,{\mathbf{z}},{\mathbf{w}}|y=1). \end{aligned}$$

Now marginalising out the variable ${\mathbf{w}}$ we get:

15 $$\begin{aligned} \sum _{{\mathbf{w}} \in {\mathbf{\mathcal {W}}}}p({\widetilde{y}_0=1},x,{\mathbf{z}},{\mathbf{w}}|y=1)&= p({\widetilde{y}_0=1}|y=1)\sum _{{\mathbf{w}} \in {\mathbf{\mathcal {W}}}}p(x,{\mathbf{z}},{\mathbf{w}}|y=1) \Leftrightarrow \nonumber \\ p({\widetilde{y}_0=1},x,{\mathbf{z}}|y=1)&= p({\widetilde{y}_0=1}|y=1)p(x,{\mathbf{z}}|y=1)\Leftrightarrow \end{aligned}$$

16 $$\begin{aligned} p({\widetilde{y}_0=1}|y=1,x,{\mathbf{z}})&= p({\widetilde{y}_0=1}|y=1) \end{aligned}$$

Furthermore in Eq. (15) by marginalising out the variable *x* we get:

17 $$\begin{aligned} \sum _{x \in \mathcal {X}} p({\widetilde{y}_0=1},x,{\mathbf{z}}|y=1)&= p({\widetilde{y}_0=1}|y=1)\sum _{x \in \mathcal {X}}p(x,{\mathbf{z}}|y=1)\Leftrightarrow \nonumber \\ p({\widetilde{y}_0=1},{\mathbf{z}}|y=1)&= p({\widetilde{y}_0=1}|y=1)p({\mathbf{z}}|y=1)\Leftrightarrow \nonumber \\ p({\widetilde{y}_0=1}|y=1,{\mathbf{z}})&= p({\widetilde{y}_0=1}|y=1) \end{aligned}$$

Thus from Eqs. (16) and (17) we can derive Eq. (14). $\square$

To prove  we need to prove that

Since the random variables ${\widetilde{Y}_0}$ and *Y* are binary it is sufficient to prove this for the two classes. For the first class we have:

$$\begin{aligned} p(x,{\widetilde{y}_0=1}|{\mathbf{z}})= & {} p(x|{\mathbf{z}})p({\widetilde{y}_0=1}|{\mathbf{z}}) \Leftrightarrow p(x|{\widetilde{y}_0=1},{\mathbf{z}}) = p(x|{\mathbf{z}}) \mathop \Leftrightarrow \limits ^{\mathrm{Lemma}~1} \\ p(x|{\widetilde{y}_0=1},{\mathbf{z}})= & {} p(x|{\mathbf{z}}) \Leftrightarrow p(x,y=1|{\mathbf{z}}) = p(x|{\mathbf{z}})p(y=1|{\mathbf{z}}) \end{aligned}$$

Using the above result for the first class, we will prove it for the second:

$$\begin{aligned}&p(x,{\widetilde{y}_0=0}|{\mathbf{z}}) = p(x|{\mathbf{z}})p({\widetilde{y}_0=0}|{\mathbf{z}}) \Leftrightarrow p(x|{\mathbf{z}}) - p(x,{\widetilde{y}_0=1}|{\mathbf{z}}) = p(x|{\mathbf{z}})(1-p({\widetilde{y}_0=1}|{\mathbf{z}})) \Leftrightarrow \\&p(x,{\widetilde{y}_0=1}|{\mathbf{z}}) = p(x|{\mathbf{z}})p({\widetilde{y}_0=1}|{\mathbf{z}}) \Leftrightarrow p(x,y=1|{\mathbf{z}}) = p(x|{\mathbf{z}})p(y=1|{\mathbf{z}}) \Leftrightarrow \\&p(x|{\mathbf{z}})-p(x,y=0|{\mathbf{z}}) = p(x|{\mathbf{z}})(1-p(y=0|{\mathbf{z}})) \Leftrightarrow p(x,y=0|{\mathbf{z}}) = p(x|{\mathbf{z}})p(y=0|{\mathbf{z}}) \end{aligned}$$

$\mathbf{Surrogate~3}~(\widetilde{Y}_1)$: Following the same methodology as we did for surrogate 2. $\square$

### Theorem 7

$\mathbf{Surrogate~2}~({\widetilde{Y}_0})$: By using the chain rule of the mutual information (Cover and Thomas 2006) the non-centrality parameter can be written as:

$$\begin{aligned} \lambda _{G(X;{\widetilde{Y}_0}|{\mathbf{Z}})} = 2NI(X;{\widetilde{Y}_0}|{\mathbf{Z}}) = 2NI(X{\mathbf{Z}};{\widetilde{Y}_0}) - 2NI({\mathbf{Z}};{\widetilde{Y}_0}) = \lambda _{G(X{\mathbf{Z}};{\widetilde{Y}_0})} - \lambda _{G({\mathbf{Z}};{\widetilde{Y}_0})}. \end{aligned}$$

Using Theorem 4, we can associate the non-centrality parameters of the G-tests $X,{\widetilde{Y}_0}$ and *X*, *Y*, so we have:

$$\begin{aligned} \lambda _{G(X;{\widetilde{Y}_0}|{\mathbf{Z}})}&= \kappa _{{\widetilde{Y}_0}}\lambda _{G(X{\mathbf{Z}};Y)} - \kappa _{{\widetilde{Y}_0}} \lambda _{G({\mathbf{Z}};Y)} = \\&=\kappa _{{\widetilde{Y}_0}} 2NI(X{\mathbf{Z}};Y) - \kappa _{{\widetilde{Y}_0}} 2NI({\mathbf{Z}};Y) = \kappa _{{\widetilde{Y}_0}} 2N(I(X{\mathbf{Z}};Y) - I({\mathbf{Z}};Y)). \end{aligned}$$

And, by using again the chain rule, the last expression can be written as:

$$\begin{aligned} \lambda _{G(X;{\widetilde{Y}_0}|{\mathbf{Z}})} = \kappa _{{\widetilde{Y}_0}} 2N I(X;Y|{\mathbf{Z}}) = \kappa _{{\widetilde{Y}_0}} \lambda _{G(X;Y|{\mathbf{Z}})}. \end{aligned}$$

$\mathbf{Surrogate~3}~(\widetilde{Y}_1)$: Following the same methodology as we did for surrogate 2. $\square$

## Footnotes

1 The software related to this paper will be available at: https://github.com/sechidis.

2 Section 6 shows how we can use our results in datasets with numerical features, while Sect. 7 suggests ways of fully extending our framework to numerical data.

3 Downloaded from http://www.bnlearn.com/bnrepository/.
